# Diversity and distribution of avian malaria and related haemosporidian parasites in captive birds from a Brazilian megalopolis

**DOI:** 10.1186/s12936-017-1729-8

**Published:** 2017-02-17

**Authors:** Carolina Romeiro Fernandes Chagas, Gediminas Valkiūnas, Lilian de Oliveira Guimarães, Eliana Ferreira Monteiro, Fernanda Junqueira Vaz Guida, Roseli França Simões, Priscila Thihara Rodrigues, Expedito José de Albuquerque Luna, Karin Kirchgatter

**Affiliations:** 1 São Paulo Zoo Foundation, Av. Miguel Estéfano 4241, São Paulo, SP 04301-905 Brazil; 2 Nature Research Centre, Akademijos 2, Vilnius, 08412 Lithuania; 3 Malaria Research Center, Superintendence for Endemic Disease Control, São Paulo, Institute of Tropical Medicine, University of São Paulo, Av. Dr. Enéas de Carvalho, Aguiar 470, São Paulo, SP 05403-000 Brazil; 4 Department of Parasitology, Institute of Biomedical Sciences, University of São Paulo, Av. Prof. Lineu Prestes 1374, São Paulo, SP 05508-900 Brazil; 5 Virology Laboratory, Institute of Tropical Medicine, University of São Paulo, Av. Dr. Enéas de Carvalho Aguiar 470, São Paulo, SP 05403-000 Brazil

**Keywords:** Avian malaria, *Plasmodium*, *Haemoproteus*, Captive birds, Zoo, Conservation

## Abstract

**Background:**

The role of zoos in conservation programmes has increased significantly in last decades, and the health of captive animals is essential to guarantee success of such programmes. However, zoo birds suffer from parasitic infections, which often are caused by malaria parasites and related haemosporidians. Studies determining the occurrence and diversity of these parasites, aiming better understanding infection influence on fitness of captive birds, are limited.

**Methods:**

In 2011–2015, the prevalence and diversity of *Plasmodium* spp. and *Haemoproteus* spp. was examined in blood samples of 677 captive birds from the São Paulo Zoo, the largest zoo in Latin America. Molecular and microscopic diagnostic methods were used in parallel to detect and identify these infections.

**Results:**

The overall prevalence of haemosporidians was 12.6%. Parasites were mostly detected by the molecular diagnosis, indicating that many birds harbour subclinical or abortive infections. In this project, birds of 17 orders (almost half of all the orders currently accepted in taxonomy of birds), 29 families, and 122 species, were tested, detecting positive individuals in 27% of bird species. Birds from the Anatidae were the most prevalently infected (64.7% of all infected animals). In all, infections with parasites of the genus *Plasmodium* (overall prevalence 97.6%) predominated when compared to those of the genus *Haemoproteus* (2.4%). In total, 14 cytochrome b (*cytb*) lineages of *Plasmodium* spp. and 2 *cytb* lineages of *Haemoproteus* spp. were recorded. Eight lineages were new. One of the reported lineages was broad generalist while others were reported in single or a few species of birds. Molecular characterization of *Haemoproteus ortalidum* was developed.

**Conclusion:**

This study shows that many species of birds are at risk in captivity. It is difficult to stop haemosporidian parasite transmission in zoos, but is possible to reduce the infection rate by treating the infected animals or/and while keeping them in facilities free from mosquitoes. Protocols of quarantine should be implemented whenever an animal is transferred between bird maintaining institutions. This is the first survey of haemosporidians in captive birds from different orders maintained in zoos. It is worth emphasizing the necessity of applying practices to control these parasites in management and husbandry of animals in captivity.

**Electronic supplementary material:**

The online version of this article (doi:10.1186/s12936-017-1729-8) contains supplementary material, which is available to authorized users.

## Background

Wild animals have been maintained in captivity since ancient Egypt, and this represented the status and power. Around eighteenth century, these animals started broadly kept in private collections, closed to general public, mostly for entertainment [1]. Through the years, these collections were gradually transformed into zoos, which since 1960s, their main concern has turned to species conservation by providing them with a healthy environment. The role of zoos in wild life conservation has markedly increased along the last decades. Nowadays, they act in environmental education, research, management, concerning animal welfare, and financing in situ conservation programmes [1, 2].

Captive environment can offer to the animals some special conditions not found in the wild, such as enough food, shelter, and protection against predators, besides veterinary care that helps them to survive various diseases [3]. However, parasitic diseases could be common and pose a greater risk in captivity as they can spread easily, leading even to death in some species [4, 5]. High density of birds is common in captivity; therefore, some species can be exposed to parasites to which they are evolutionary non-adapted and have no competent immune response against such infections [6].

Birds can be affected by many different blood parasites, some of them are nematodes, like microfilaries, but protozoan parasites can also be found infecting these animals, such as *Trypanosoma* and *Babesia* species, among others [4]. Probably the parasites with the major importance in birds are species of the order Haemosporida (phylum Apicomplexa), composed of four families: Plasmodiidae, Haemoproteidae, Leucocytozoidae and Garniidae, and of four genera, *Plasmodium*, *Haemoproteus, Leucocytozoon* and *Fallisia* [7]. These parasites have heteroxenous life cycles, and are transmitted exclusively by blood-sucking dipterans [7]. Haemosporidians have been reported in many zoos around the world. In Japan, *Plasmodium (Bennettinia) juxtanucleare* was responsible for the death in a white eared-pheasant (*Crossoptilon crossoptilon*) [8]. In Brazil, *Plasmodium* spp. were detected in 36% of captive psittacine birds in three zoological gardens [9], and *Plasmodium (Novyella) nucleophilum* was identified in an Egyptian Goose (*Alopochen aegyptiaca*) that died in São Paulo Zoo [10]. In Europe, *Haemoproteus minutus* was reported causing death in parrots [11, 12]. In USA, *Plasmodium* sp. was diagnosed in asymptomatic Chilean flamingos in a zoo in Chicago [13] and *Plasmodium* sp. and *Haemoproteus* sp. were responsible for death in a zoo in Texas [14]. In Italy, *Plasmodium* sp. was detected in raptors [15]. Finally, some species of *Plasmodium* are well known to cause mortality in penguins in captive and rehabilitation centres all over the world [16–18]. Penguins are considered to be one of the most sensitive bird groups to *Plasmodium* infections, particularly young birds with a naïve immune system [7, 16–18].

To diagnose haemosporidian infections, morphological features of the parasites encountered in blood film are analysed. However, more recently, molecular techniques are also used to confirm the morphological analysis and to provide more taxonomy information [4, 6]. Due to the high sensitivity of molecular techniques, parasite DNA can be found when gametocytes are not observed [11, 14]. When this happens, may be a clue of partial or abortive (ectopic) development of haemosporidian parasites. In this case, the initial parasite development occurs (tissue stages in birds or initial sporogonic stages in dipteran insects develop), but the parasites cannot complete their life cycles [7, 12].

Neotropical regions are considered hotspots of avian diversity but there are still few bird parasites studies performed in these locations, and many new haemosporidian species likely to be discovered [19]. Sampling of animals in zoos is easier and less costly than collecting samples of free living animals, with the additional advantage of following them during a long period of time in order to keep their medical history. The objectives of this research were (1) to determine distribution and identity of the lineages of malaria parasites (*Plasmodium* spp.) and phylogenetically related *Haemoproteus* spp. in São Paulo Zoo, (2) to verify if there is some seasonality in the parasite prevalence, (3) to suggest some practical solutions to minimize parasite transmission.

## Methods

### Study site

This study was performed in the São Paulo Zoo (23°39′S, 46°37′W). The park, opened in 1958 within the largest city in Brazil, São Paulo, is currently recognized as the biggest zoo in South America, harbouring approximately 3000 animals. São Paulo Zoo is located inside a state park (Parque Estadual das Fontes do Ipiranga/PEFI), in an Atlantic Forest remnant. In this area, the Ipiranga stream creates small lakes where many captive, wild and migratory birds live together. In winter months, the place hosts migratory birds from different American countries and other Brazilian regions.

### Population studied

**Fig. 1:**
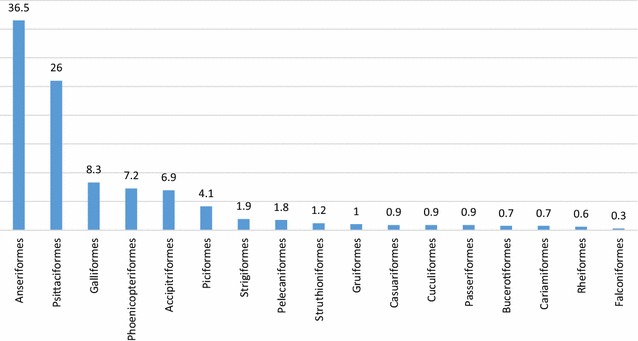
Population studied. Orders of examined birds are presented in decreasing direction of abundance, and they were categorized in highly abundant, moderately abundant, and rare. Exact numbers of all examined species are given in Table 1 and Table S1. The ordinate shows the relative order abundance (in percentage)

In all, 1254 blood samples were collected from 677 captive birds belonging to 122 species, 29 families and 17 orders. The tested individuals represented 42.1% of all captive birds from São Paulo Zoo [20]. Species of Anseriformes and Psittaciformes were particularly extensively sampled, comprising together 62.5% of all analysed samples (36.5 and 26%, respectively) (Fig. 1). Species of Galliformes, Phoenicopteriformes, Accipitriformes and Piciformes were less frequently sampled and together represented 26.6% of analysed samples. A third group (rarely sampled birds) contained representatives of 11 different orders (10.9% of all samples).

In regard to number of samples from different avian families, the birds of the Anatidae and Psittacidae were best sampled (36.3 and 25.7% of all samples, respectively). The birds moderately sampled were: Phoenicopteridae (7.2% of all samples), Accipitridae (5.9%), Phasianidae (5.2%), Ramphastidae (4.1%), Cracidae (2.7%), Strigidae (1.5%), Threskiornitidae (1.3%), Sthruthionidae (1.2%) and Cathartidae (1%). Samples from birds of 18 families represented < 1% of all samples collected during this study; these were Casuaridae, Gruidae, Musophagidae, Cariamidae, Bucorvidae, Rheidae, Odontophoridae, Pelecanidae, Tytonidae, Cacatuidae, Cotingidae, Falconidae, Sturnidae, Anhimidae, Bucerotidae, Icteridae, Rallidae, Thraupidae.

Among sampled animals, 41.5% were males, 40.5% females, and in 18% of samples bird gender was not possible to be determined. The majority of the animals (87.4%) were adults. In the course of this study, some individual birds were tested several times. In all, 262 animals (38.7%) were sampled at least two times during this study, but there were individuals, from which up to 15 samples were collected.

### Blood collection

Samples from captive birds were collected between December 2011 and June 2015. All blood samples were collected for routine veterinary examinations from animals presenting or not presenting clinical signs of disease, or during preventive examinations. The blood sampled collected from animals during preventive procedures represented about a third of all samples collected during this study. The venous blood was collected from brachial, metatarsal or jugular veins. Two thin blood smears were prepared as described [21]. The remaining blood was placed in a lithium heparin tube and gently homogenized for preventing blood clotting. In the lab, the samples were centrifuged and the erythrocytes were kept on −20 °C until the DNA extraction.

### Microscopic examination

The thin blood smears were fixed by 100% methanol in the same day of collection and stained with a 10% Giemsa work solution for 1 h, then examined microscopically for 20–25 min with 100 fields viewed at low magnification (400×) and 100 fields at high magnification (1000×) [7], using an Olympus BX41 light microscope. The intensity of parasitaemia was determined by actual counting the number of parasites per 1000 erythrocytes, as recommended [22].

### Genomic DNA extraction

DNA from blood samples was extracted with the Wizard^®^ SV 96 Genomic DNA Purification System (Promega) as described [21]. Briefly, 10 μl of red blood cell pellets was completed to 200 μl with ultrapure water and an initial lysis was performed with Proteinase K. Whole Blood Lysis Buffer (400 μl) was added and incubated overnight at room temperature. The lysates were transferred into the columns and washed according the manufacturer’s instructions. DNA was eluted in 100 μl of Nuclease-FreeWater and stored at −20 °C.

### PCR amplification of mitochondrial cytochrome b fragments, sequencing and sequence data analysis

Polymerase chain reactions (PCR) were conducted using a nested PCR targeting the mitochondrial cytochrome b (*cytb*) gene of *Haemoproteus* and *Plasmodium* species [23]. The first reaction used primers HaemNFI/HaemNR3 and 50 ng genomic DNA (gDNA). In the nested reaction, performed with a second pair of primers (HaemF/HaemR2), 1 µl of the product from the first reaction was used as a template. In each PCR, three controls were carried out in parallel: two samples, presenting different *Plasmodium* spp. parasitaemias (<0.01 and 6.49%), and ultrapure water, as a negative control.

PCR products were sequenced by Big Dye Terminator v3.0 Cycle Sequencing Kit in ABI Genetic Analyzer (ABI, USA), using PCR oligonucleotides (HaemF and HaemR2). *Cytb* sequences of ~480 bp were obtained and used in this study. The found sequences were aligned with sequences from the MalAvi database [24] in order to verify if the sequences were new lineage sequences. The sequences possessing at least one different nucleotide were considered unique lineages and were named according to the MalAvi nomenclature [24] and deposited in GenBank and MalAvi.

### Phylogenetic analysis

The phylogenetic relationship among reported parasites was inferred using *cytb* gene sequences. GenBank accessions of the used sequences are given in phylogenetic trees. The phylogenetic reconstruction was performed separately for *Haemoproteus* and *Plasmodium* using the Bayesian inference method implemented in MrBayes v3.2.0 [25]. Bayesian inference was executed with two Markov Chain Monte Carlo searches of 3 million generations, each with sampling of 1 in 300 trees. After a burn-in of 25%, the remaining 15,002 trees were used to calculate the 50% majority-rule consensus tree. The phylogeny was visualized using FigTree version 1.4.0 [26].

Additionally, median-joining phylogenies were generated using Network software version 4.6 [27] with default parameters and transversions weighted two times as much as transitions. This analysis was carried out using 865 *cytb* sequences of 41 *Plasmodium* (*Haemamoeba*) spp. lineages, with 467 bp each sequence. These sequences were selected from MalAvi database due to their similarity of ≥96% with the sequences of *Plasmodium* (*Haemamoeba*) spp. lineages obtained in this study.

### Seasonality

In order to determine whether there was some seasonality in infection prevalence, each new case reported was analysed according to the date of detection and classified under the season: spring (October to December), summer (January to March), autumn (April to June) and winter (July to September). The seasonality analysis was done considering one sample by individual (the first one collected). Data from all years were combined. To verify if climatic factors may have influenced the analysis, the average precipitation and minimum average temperature data recorded during this study were examined. The data were obtained from the weather station of Meteorological Institute of Astronomy, Geophysics and Atmospheric Sciences of São Paulo University, located in front of São Paulo Zoo [28].

### Statistical analysis

Statistical analysis was performed using SPSS (Statistical Package for the Social Sciences) for Windows, version 15.0 (SPSS Inc., Chicago, IL, USA) and Microsoft Excel 2010. To assess the effects of five independent variables (Family, season, gender, age and captive time) on haemosporidian prevalence, the Chi square test, Fisher’s exact, or likelihood ratio tests were used. A significance level of *p* < 0.05 was used.

## Results

### Prevalence of infections

In all, 85 bird individuals (12.6%) were positives for *Plasmodium* and *Haemoproteus* spp. (Table 1). Infected birds belonged to 11 orders (64.7% of all orders tested), 14 families (48.3% of all families tested) and 33 species (27% of all species tested). The orders with the majority of infected animals were Anseriformes (64.7% of all infected animals), followed by Galliformes (11.8% of all infected animals); Accipitriformes, Phoenicopteriformes and Piciformes (each with 3.5% of all infected animals); Psittaciformes (5.9% of all infected animals), Passeriformes (2.4% of all infected animals); and Cuculiformes, Gruiformes, Pelecaniformes and Struthioniformes (each with 1.2% of all infected animals). The greatest prevalence of *Plasmodium* and *Haemoproteus* infections was reported in species of the Anatidae (64.7% of all infected animals), followed by species of the Cracidae, Phasianidae and Psittacidae (5.9% of all infected animals of each family). Belonging to the Anatidae was significantly associated with high prevalence of these infections (*p* < 0.05).

**Table 1:** Birds with positive results of PCR-based diagnostics of *Plasmodium* and *Haemoproteus* parasites and their lineages

ORDER Family	*Host species* (Common name)	Birds (positives)	Samples (positives)	Parasites and lineages	Genbank accession
Accipitriformes					
Accipitridae	*Buteogallus urubitinga* (Great Black-Hawk)	1 (1)	6 (1)	*Plasmodium* sp. PESA01	EU684543
Cathartidae	*Sarcoramphus papa* (King Vulture)	6 (2)	10 (3)	*Plasmodium* sp. NYCNYC01	KU057967
Anseriformes					
Anatidae	*Alopochen aegyptiaca* (Egyptian goose)^∆^	10 (5)	18 (7)	*Plasmodium nucleophilum* DENPET03 *Plasmodium* sp. DENVID01 *Plasmodium* sp. NYCNYC01	AY640137 KU057966 KU057967
	*Amazonetta brasiliensis* (Brazilian Teal)	4 (1)	8 (1)	*Plasmodium* sp. NYCNYC01	KU057967
	*Anser cygnoides* (Swan goose)**^∆^	1 (1)	4 (4)	*Plasmodium* sp. NYCNYC01 *Plasmodium* sp. PADOM09	KU057967 AF069611
	*Cereopsis novahollandiae* (Cape Barren goose)^∆^	12 (1)	19 (1)	*Plasmodium* sp. **CERNOV01**	KX171623
	*Coscoroba coscoroba* (Coscoroba swan)	24 (1)	39 (1)	*Plasmodium elongatum* GRW06	DQ368381
	*Cygnus atratus* (Black swan)^∆^	123 (32)	175 (53)	*Plasmodium nucleophilum* DENPET03 *Plasmodium* sp. DENVID01 *Plasmodium elongatum* GRW06 *Plasmodium* sp. MYCAME02 *Plasmodium* sp. NYCNYC01 *Plasmodium* sp. PESA01	AY640137 KU057966 DQ368381 JX546135 KU057967 EU684543
	*Cygnus melanocoryphus* (Black-necked swan)	31 (3)	75 (5)	*Plasmodium* sp. NYCNYC01 *Plasmodium* sp. PESA01	KU057967 EU684543
	*Dendrocygna viduata* (White-faced Whistling Duck)	4 (1)	5 (1)	*Plasmodium* sp. NYCNYC01	KU057967
	*Netta erythrophthalma* (Southern Pochard)	2 (1)	2 (1)	*Plasmodium nucleophilum* DENPET03	AY640137
	*Plectropterus gambensis* (Spur-winged goose)^∆^	7 (1)	16 (1)	*Plasmodium* sp. NYCNYC01	KU057967
	*Tadorna ferruginea* (Ruddy Shelduck)^∆^	10 (7)	28 (10)	*Plasmodium* sp. NYCNYC01 *Plasmodium* sp. DENVID01	KU057967 KU057966
	*Tadorna variegata* (Paradise shelduck)^∆^	1 (1)	5 (4)	*Plasmodium* sp. NYCNYC01	KU057967
Cuculiformes					
Musophagidae	*Musophaga violacea* (Violet Turaco)^∆^	2 (1)	3 (1)	*Plasmodium* sp. SPMAG06	HM031936
Galliformes					
Cracidae	*Mitu tomentosum* (Crestless curassow)*	5 (1)	10 (1)	*Plasmodium* sp. **MITOM01**	KX171625
	*Nothocrax urumutum* (Nocturnal curassow)	5 (1)	9 (1)	*Plasmodium* sp. **NOTURU01**	KX171626
	*Penelope obscura* (Dusky-legged guan)	2 (1)	2 (1)	*Haemoproteus ortalidum* **PENOBS01**	KX171627
	*Pipile jacutinga* (Black-fronted piping guan)***	3 (2)	9 (3)	*Plasmodium* sp. NYCNYC01 *Plasmodium nucleophilum* DENPET03	KU057967 AY640137
Phasianidae	*Pavo cristatus* (Blue peafowl)^∆^	31 (4)	53 (5)	*Plasmodium elongatum* GRW06 *Plasmodium* sp. DENVID01	DQ368381 KU057966
	*Pavo muticus* (Green peafowl)***^∆^	4 (1)	7 (3)	*Plasmodium* sp. DENVID01	KU057966
Gruiformes					
Rallidae	*Aramides cajaneus* (Grey-necked wood rail)	1 (1)	2 (1)	*Plasmodium* sp. **ARACAJ01**	KX171622
Passeriformes					
Icteridae	*Psarocolius decumanus* (Crested oropendola)	1 (1)	1 (1)	*Plasmodium nucleophilum* DENPET03	AY640137
Thraupidae	*Saltator atricollis* (Black-throated saltator)	1 (1)	1 (1)	*Plasmodium* sp. **SALAT01**	KX171629
Pelecaniformes					
Threskiornithidae	*Eudocimus ruber* (Scarlet ibis)	6 (1)	7 (1)	*Haemoproteus* sp. **EUDRUB01**	KX171624
Phoenicopteriformes					
Phoenicopteridae	*Phoenicopterus chilensis* (Chilean flamingo)*	34 (3)	39 (4)	*Plasmodium* sp. **MITOM01** *Plasmodium nucleophilum* DENPET03 *Plasmodium* sp. MYCAME02	KX171625 AY640137 JX546135
Piciformes					
Ramphastidae	*Ramphastos toco* (Toco Toucan)	4 (2)	12 (3)	*Plasmodium nucleophilum* DENPET03 *Plasmodium* sp. NYCNYC01 *Plasmodium* sp. **RAMVIT01**	AY640137 KU057967 KX171628
	*Ramphastos vitellinus* (Channel-billed toucan)**	2 (1)	7 (2)	*Plasmodium* sp. **RAMVIT01**	KX171628
Psittaciformes					
Psittacidae	*Amazona aestiva* (Blue-fronted amazon)	12 (1)	35 (1)	*Plasmodium* sp. NYCNYC01	KU057967
	*Anodorhynchus hyacinthinus* (Hyacinth macaw)**	22 (2)	46 (2)	*Plasmodium* sp. NYCNYC01	KU057967
	*Cyanopsitta spixii* (Spix’s macaw)****	4 (1)	8 (1)	*Plasmodium* sp. NYCNYC01	KU057967
	*Guarouba guarouba* (Golden parakeet)**	7 (1)	12 (1)	*Plasmodium nucleophilum* DENPET03	AY640137
Struthioniformes					
Struthionidae	*Struthio camelus* (Common ostrich)^∆^	8 (1)	12 (1)	*Plasmodium* sp. NYCNYC01	KU057967

IUCN Threatened classification: (*) near threatened, (**) vulnerable, (***) endangered, (****) critically endangered. Other species are classified as Least Concern. ^∆^ Not native to Brazil

All sampled animals belonging to 15 families were free of haemosporidian infections; these were species of the Anhimidae (Anseriformes), Bucerotidae and Bucorvidae (Bucerotiformes), Falconidae (Falconiformes), Odonthophoridae (Galliformes), Cariamidae and Gruidae (Gruiformes), Cotingidae and Sturnidae (Passeriformes), Pelecanidae (Pelecaniformes), Cacatuidae (Psittaciformes), Rheidae (Rheiformes), Strigidae and Tytonidae (Strigiformes), Casuaridae (Casuariformes). All negative records are shown in the Additional file 1.

In all, 33 bird species were positive for *Plasmodium* or *Haemoproteus* parasites. The majority of them (63.6%) can be found in wildlife in Brazil (Table 1). It is important to note that three species have been classified as threatened according to IUCN [29]; these are *Cyanopsitta spixii,* considered critically endangered, and *Pipile jacutinga* and *Pavo muticus,* considered threatened (Table 1).

The infected animals represented 13.3% of all adults analysed, 7.1% of the all young birds tested, 11.7% of all female and 9.6% of all male of the study. There were no significant differences in prevalence of *Haemoproteus* and *Plasmodium* infections regarding gender or age. Regarding the time spent in captivity in São Paulo Zoo, 62 (72.9%) of infected birds lived in São Paulo Zoo for more than 10 years, 16 (18.8%) between 5 and 10 years, 5 (5.9%) between 1 and 5 years and 2 (2.4%) for less than 1 year, after they were sampled. The captive time of more than 10 years in São Paulo Zoo was significantly associated with the presence of haemosporidian infections (*p* < 0.005). During the study period, 39 (45.9%) of parasite positive animals died, 1 (1.2%) was considered as disappeared, 3 (3.5%) were transferred for other institutions, and 42 (49.4%) were alive at the end of the period.

The origin of parasite positive animals was also analysed: 57 (67.1%) birds were born in the São Paulo Zoo, 9 (10.6%) came from other institutions of São Paulo State, 7 (8.2%) were donated to São Paulo Zoo, 7 (8.2%) came from other Brazilian states (Maranhão, Pernambuco, Paraná, Rondônia and Rio Grande do Sul), 3 (3.5%) were seized by government and 2 (2.3%) came from another countries (Philippines and Germany).

### Seasonal variation in infection prevalence

**Fig. 2:**
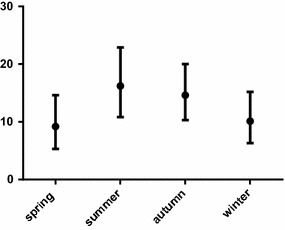
Overall prevalence of *Plasmodium* and *Haemoproteus* infections in birds from São Paulo Zoo in different seasons. Total number of examined birds (n) was 677. Data for all years were combined. The ordinate is the prevalence of infection (in percentage). *Vertical lines* are 95% confidence limits

In order to check the presence of seasonality in infection prevalence, each new reported case was analysed according the date of detection. Percentage of positive birds was determined during each season. Although greater number of infected birds was detected during summer sampling, no significant prevalence variation in different seasons was found (Fig. 2).

### Morphological, molecular and phylogenetic analysis

In all, 1230 samples were processed with molecular techniques (98.1%). In remaining 24 samples (1.9%), only blood smears were available for analysis, and in all of them parasite were not seen. PCR was positive in 127 samples (10.3%), and parasites were detected in 62 samples (48.8%) after microscopic examination of blood films. All samples with PCR negative results were also negative by microscopy.

**Fig. 3:**
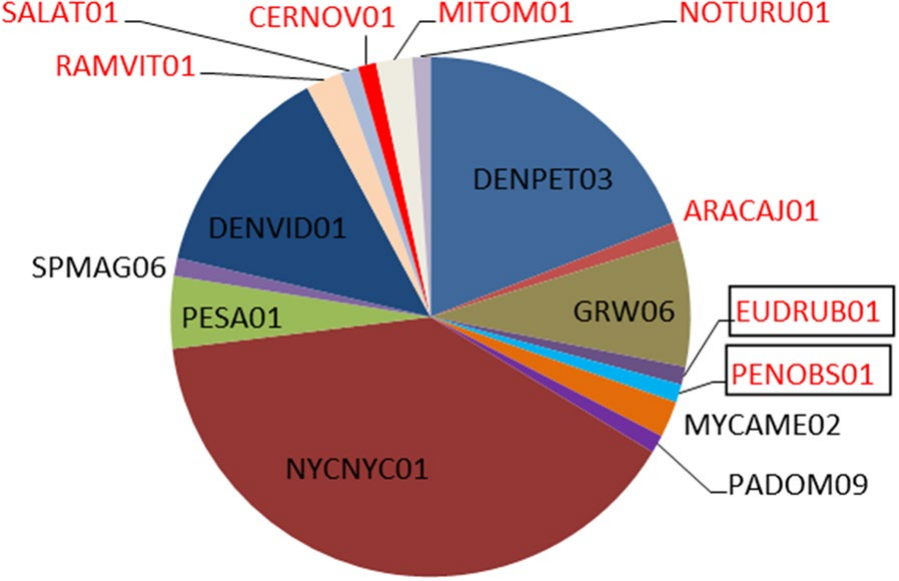
*Plasmodium* and *Haemoproteus* parasite lineage diversity (in percentage) in relation to the total number of detected lineages. *Red font* indicates new lineages. *Haemoproteus* lineages are boxed

A total of 16 different haemosporidian lineages were found in 85 birds (Table 1; Fig. 3). Fourteen *Plasmodium* spp. lineages (87.5% of all reported lineages) were detected in 83 individuals and two *Haemoproteus* spp. lineages were found in two animals. Eight new lineages were found in this study: 6 belong to *Plasmodium* spp. (pRAMVIT01, pMITOM01, pARACAJ01, pSALAT01, pCERNOV01 and pNOTURU01) and 2 came from *Haemoproteus* spp. (hPENOBS01 and hEUDRUB01).

The lineage pDENPET03 of malaria parasite of *P. nucleophilum* was found in 17 samples of birds belonging to 8 different species of 6 families (Table 1). Microscopic examination of blood films confirmed the nucleophilic pattern of blood stages of this parasite, and all the other main characters, which are characteristics of in this species (see [10]).

The pGRW06 lineage was found in 7 bird individuals belonging to 2 species of Anseriformes and one species of Galliformes (Table 1). It has been already identified as *Plasmodium (Huffia) elongatum*, a widespread species of malaria parasite, which trophozoites and meronts develop in young erythrocytes and gametocytes are present only in mature red blood cells. These readily visible morphological features were confirmed in all positive blood smears from these birds.

The pMYCAME02 lineage of *Plasmodium* sp. was described in two individuals, *Cygnus atratus* and *Phoenicopterus chilensis* (Table 1), but blood smears from these birds were negative. The pMITOM01 lineage of *Plasmodium* sp. was found in *Mitu tomentosum* and *Phoenicopterus chilensis* (Table 1), but the only positive blood smear of these animals presented very light parasitaemia (a few growing blood stages seen), making impossible the morphological parasite species identification. The pPADOM09 lineage of *Plasmodium* sp. was molecularly detected in *Anser cygnoides* (Table 1). This individual had also 3 samples found positive for pNYCNYC01 lineage, but the slides showed very light parasitaemia and morphological identification was impossible. The pSPMAG06 lineage of *Plasmodium* sp. was found in a PCR positive sample from *Musophaga violacea* (Table 1), with negative blood smear.

**Fig. 4:**
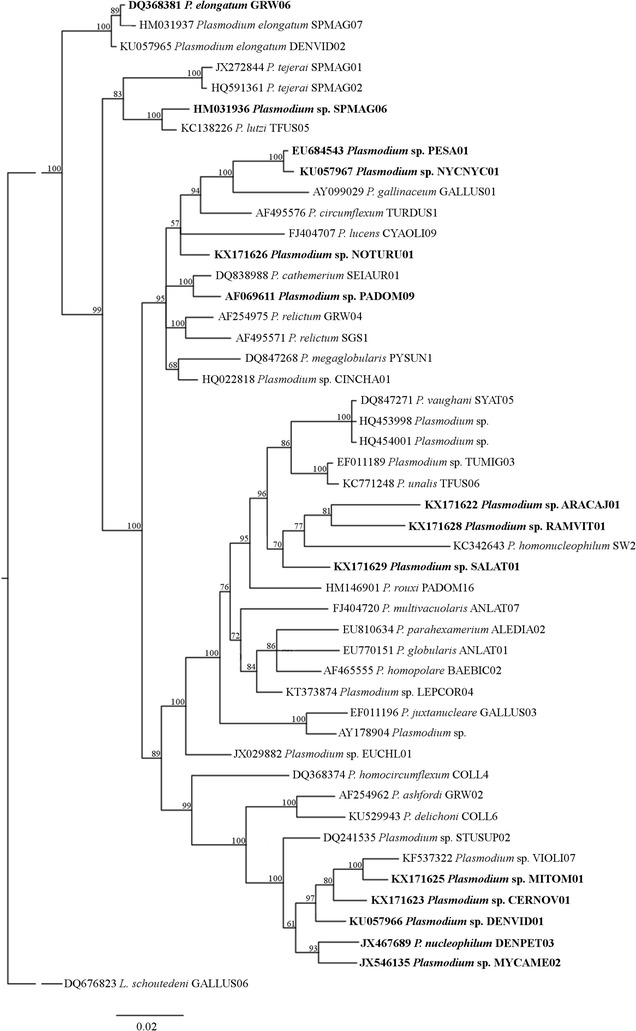
Bayesian phylogeny of cytochrome b gene lineages of avian *Plasmodium* species. Lineages recorded in this work are given in *Bold*. Codes of the lineages are given after species names of parasites, and GenBank accession numbers are provided before the parasite species names. Nodal support values (in percentage) indicate posterior clade probabilities

Of all the *Plasmodium* lineages found, 8 were clustered together in two clades containing malaria parasites of subgenus *Novyella* (Fig. 4). The first group presented the lineages pRAMVIT01, pARACAJ01 and pSALAT01 that are closed related to *Plasmodium homonucleophilum*. The second group was formed by the lineages pCERNOV01, pMITOM01, pDENPET03, pDENVID01 and pMYCAME02. There were 5 lineages that clustered with *Haemamoeba* subgenus: pNYCNYC01, pPESA01, pNOTURU01, pPADOM09 and pSPMAG06. The lineage pGRW06, which belongs to species of *Huffia*, was separated from all the other subgenera (Fig. 4).

Among the 62 positive blood smears, 12 co-infections (19.4%) were identified. Molecular diagnosis did not read these co-infections, since only one sequence was obtained per sample using our methodology. *Plasmodium* and *Haemoproteus* co-infections were visualised in 11 samples, which all were obtained from *Cygnus atratus* and with the same lineage detected (the lineage pNYCNYC01 of *Plasmodium*). In one co-infection, observed in *Saltator atricolis,* two *Plasmodium* species were found during the microscopic examination, but only the pSALAT01 lineage was detected by PCR.

**Fig. 5:**
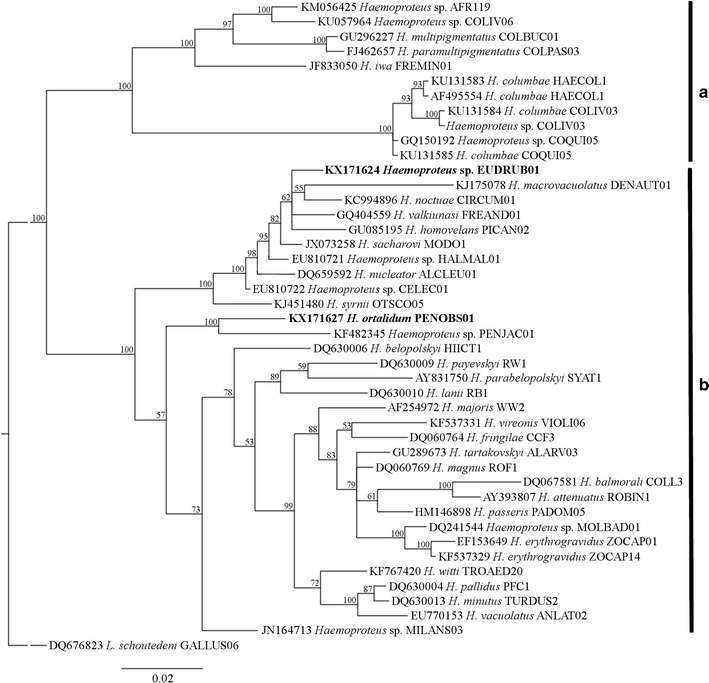
Bayesian phylogeny of cytochrome b gene lineages of *Haemoproteus* species. Lineages recorded in this work are given in Bold. Codes of the lineages are given after the species names of parasites, and GenBank accession numbers are provided before the parasite species names. Nodal support values (in percentage) indicate posterior clade probabilities. Vertical bars indicate clades of species of subgenus *Haemoproteus* (**a**) and *Parahaemoproteus* (**b**)

The *Haemoproteus* lineages, hEUDRUB01 and hPENOBS01, were present in *Eudocimus ruber* and *Penelope obscura*, respectively (Table 1). The *Haemoproteus* phylogenetic tree formed two well supported clades, clearly separating species of *Parahaemoproteus* and *Haemoproteus* subgenera (Fig. 5). Based of phylogenetic analysis, all the *Haemoproteus* sequences found in captive animals belonged to *Parahaemoproteus* subgenus because they were clustered in the clade containing these parasites.

Interestingly, two individuals were positive for different lineages along the study: one of them, *Anser cygnoides,* had 4 samples screened, with the pNYCNYC01 lineage detected in 3 samples and the pPADOM09 lineage in one sample; the other one, *Ramphastos toco,* had 5 samples screened, but only two of them were positive, one with the pNYCNYC01 lineage and one with the pDENPET03 lineage.

### Parasite genetic lineages and morphological considerations

**Fig. 6:**
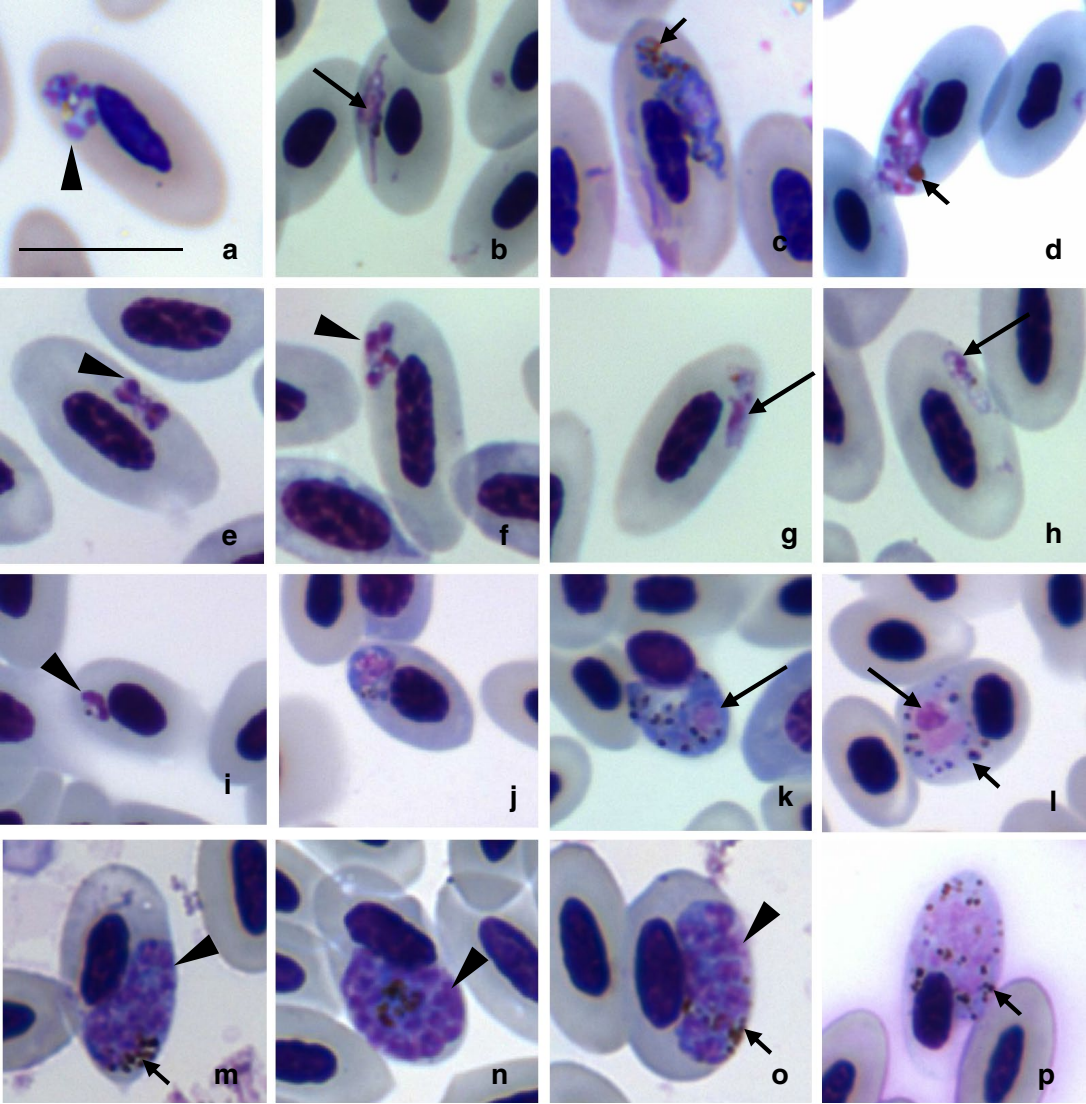
Blood stages of four *Plasmodium* spp. found in this study. The lineage pDENVID01 (**a**–**d**): note small meronts possessing a retractable globule (**a**), growing meronts possessing outgrowth (**b**) and mature macrogametocytes (**c**) and microgametocytes (**d**) with amoeboid outline; microgametocytes possess big haemozoin pigment granules. The lineage pRAMVIT01 (**e**–**h**): note small meronts and retractable globules (**e**, **f**), and gametocytes usually found in polar and subpolar position in infected cell and possessing distinct prominent nuclei (**g**, **h**). The lineage pSALAT01 (**i**–**l**): it was identified by phylogenetic analysis as a *Novyella* lineage, but the infected host had mixed infection with another *Plasmodium* lineage; small meronts (**i**), a feature of *Novyella* subgenus and growing meronts (**j**) and gametocytes (**k**, **l**) with *Haemamoeba* morphological characteristics. The lineage pNYCNYC01 (**m**–**p**): phylogenetic analysis has identified it a lineage of *Haemamoeba* subgenus; it has meronts with prominent cytoplasm and they displace the nucleus of infected erythrocytes (**m**–**o**), the same characteristic can be seen in mature microgametocytes (**p**). *Scale bar* 10 µm. *Triangle* merozoites. *Long arrow* parasite nuclei. *Small arrow* haemozoin pigment. *Arrow head* merozoite

The lineage pDENVID01 was found only in birds of the Anatidae and Phasianidae; it was reported in 5 bird species and 12 animals (Table 1). Morphologically, the parasite of this lineage has features of malaria parasite species belonging to subgenus *Novyella*: erythrocytic meronts possess scanty cytoplasm, and retractable globules (Fig. 6a) often seen. Growing meronts possess outgrowth (Fig. 6b). Mature erythrocytic meronts contain between 5 and 7 merozoites. Mature gametocytes are elongated, markedly amoeboid in outline; pigment granules can be grouped together forming a group (Fig. 6c) or bigger clamp (Fig. 6d), which is particularly evident in microgametocytes, while in macrogametocytes, such clamps were uncommon (Fig. 6c). One individual of *Cereopsis novahollandiae* had a sequence with 98% of similarity with the lineage pDENVID01, and this new lineage was named as pCERNOV01. However, the lineage pCERNOV01 was not identified to species level nor morphologically evaluated because of light parasitaemia and, particularly due to absence of mature gametocytes in available blood films.

The lineage pRAMVIT01 was found infecting only species of Ramphastidae (*Ramphastos toco* and *Ramphastos vitellinus*) (Table 1), and probably it is specific to birds of this family. Blood stages of this parasite are morphologically similar to *Plasmodium rouxi,* with small meronts containing retractable globules (Fig. 6e, f), but can be distinguished from the latter species due to morphology of its gametocytes, which possess dense prominent nuclei with regular outline (Fig. 6g, h). Meronts of this parasite assume mainly a polare position in erythrocytes, possess not so well evident globules as in *P. rouxi*, which is particularly evident in completely mature meronts (Fig. 6e–h). The genetic similarity found between pRAMVIT01 and pPADOM16 (GenBank #HM146901) from *P. rouxi* was 94%, indicating that pRAMVIT01 could be a new species, but more data are needed for better characterization of its blood stages.

The lineage of pSALAT01 was identified only in an individual of *Saltator atricolis* (Table 1), with relatively high parasitaemia (2.4%). Its sequence clustered together with other *Novyella* species, but when smears were analysed, presence of a mixed infection composed by two *Plasmodium* species was determined (Fig. 6i–l). One *Plasmodium* species is similar to *Novyella* species: it characterized by small erythrocytic meronts with scanty cytoplasm. Due to high parasitaemia of *Novyella* parasites, it is probable that PCR protocol detected sequence if this infection. In parallel, another *Plasmodium* species was present. The latter parasite has large roundish both gametocytes and meronts, which markedly displaced the nuclei of the infected erythrocytes, the features of *Haemamoeba* species. However, DNA sequence of this parasite was not detected using molecular protocols established in this study.

The lineage pNYCNYC01 was found in 16 bird species (54 samples) from 6 orders (Table 1). About 42% of samples contained mostly low parasitaemia (<0.01%), and it was difficult to identify parasite species. However, we detected *Plasmodium* sp. and *Haemoproteus* sp. mixed infections in 47.8% of positive blood smears; all these samples were from birds of the Anatidae. Parasites of this lineage possess meronts with plentiful cytoplasm (Fig. 6m–o); nuclei of infected erythrocytes are displaced by mature parasites (Fig. 6m–p), but might be not displaced by developing young meronts; gametocytes were mainly roundish (Fig. 6p). This lineage has 99% of similarity with the lineage pPESA01, which was found in this study infecting 4 individuals from the Anatidae and Accipitridae (Table 1), but parasitaemia was observed only in one sample. Unfortunately, morphological identification of this parasite was impossible in either case due to the lack of preparations containing all blood stages and/or the presence of co-infections with *Haemoproteus*.

**Fig. 7:**
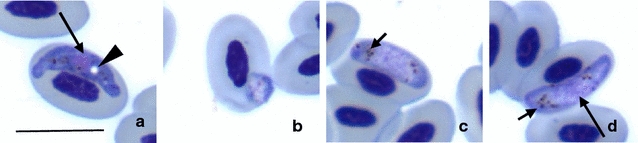
Gametocytes of *Haemoproteus (Parahaemoproteus) ortalidum* (lineage hPENOBS01, GenBank KX171627) from the blood of Dusky-legged guan (*Penelope obscura*). Note the elongate macrogametocyte (**a**) possessing a large round vacuole, which might reach 3 μm in diameter, roundish young microgametocytes (**b**) and elongate mature microgametocytes (**c**, **d**). *Arrow* vacuole. *Scale bar* 10 µm. *Long arrow* parasite nuclei. *Arrow head* vacuole. *Small arrow* haemozoin pigment

Parasites of the lineage hPENOBS01 had both elongate and roundish gametocytes (Fig. 7a–d) possessing large (about 1–3 μm in diameter) and clear circular vacuoles (Fig. 7a) characteristic of *Haemoproteus (Parahaemoproteus) ortalidum*.

### Haplotype network

**Fig. 8:**
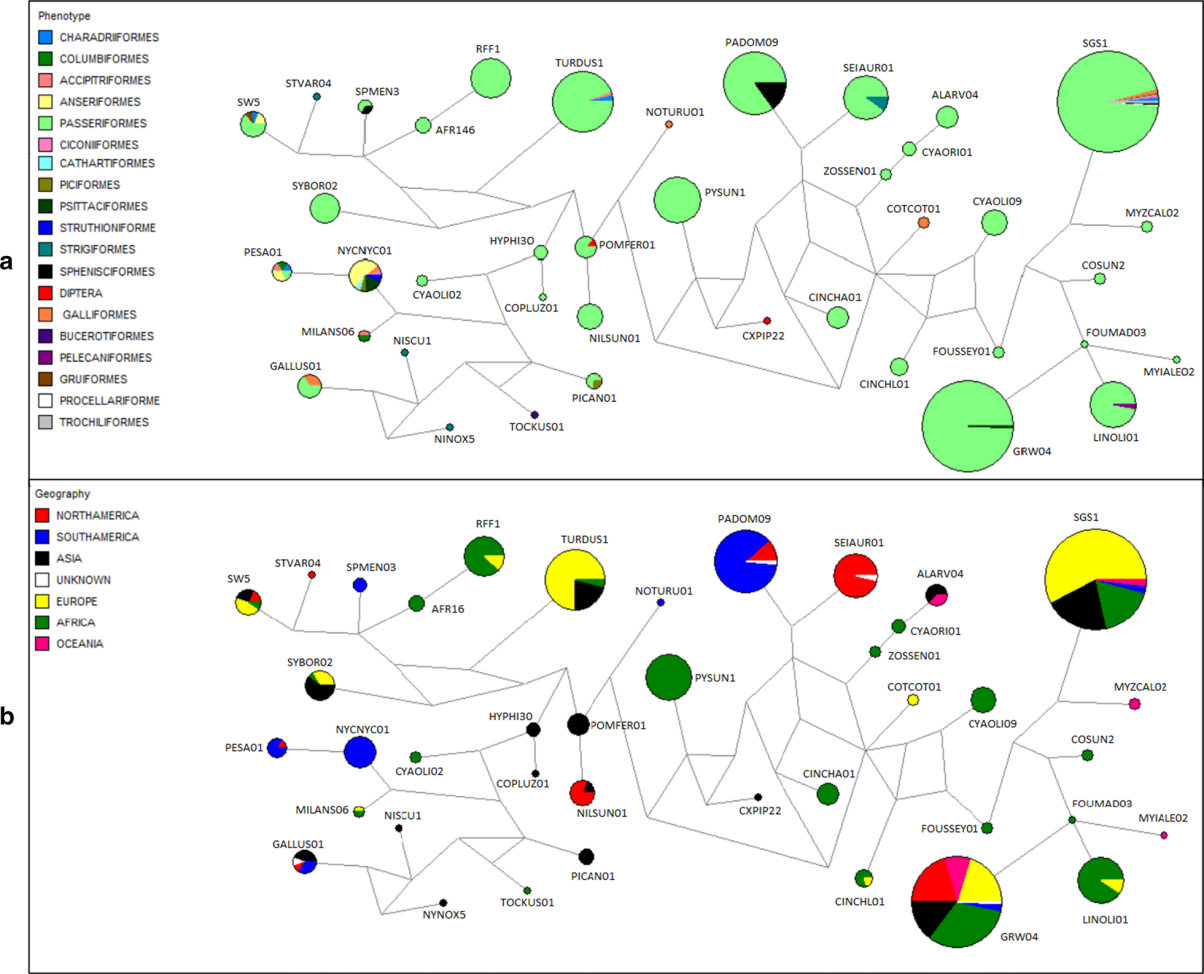
Median-joining network of a worldwide collection of *Plasmodium* (*Haemamoeba)* parasite *cytb* haplotypes. *Circles* represent haplotypes, and their sizes are proportional to haplotype frequencies.* Colours *indicate the host order (**a**) or region of origin of the samples (**b**). Each line connecting the *circles* represents a mutational step

To better understand the distribution of haplotypes of *Plasmodium* (*Haemamoeba)* sp. lineages, two networks were built with the sequences obtained in this study and those available in the MalAvi database. In the first (Fig. 8a), the lineages were classified according to their reports in hosts by avian order. The results showed the dominance of sequences described in species of Passeriformes, with some lineages being detected only in birds of this order. A few lineages were observed in birds of more than one order. The pNYCNYC01 and pPESA01 lineages showed a limited geographic distribution (Fig. 8b), being reported only in South and North Americas. In fact, the network built according to the geographic region, in which the certain sequences have been described, showed that the lineages found in the South America were also detected in North America, but scarcely in other geographic regions, except for the lineages pSGS1 and pGRW04 of *Plasmodium relictum,* which are of broad both the host and geographical distribution (Fig. 8b). In terms of mutational steps, the pNYCNYC01 and pPESA01 lineages were closer to lineages described in Asia than other geographic regions (Fig. 8b).

## Discussion

**Fig. 9:**
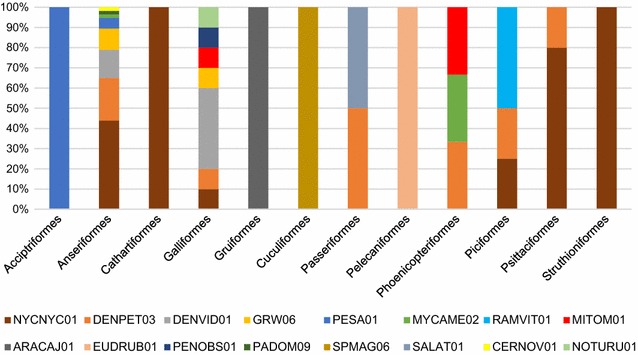
Lineage diversity (in *percentage*) of reported parasites by birds of different orders. The ordinate shows percentage

Avian malaria causing by species of *Plasmodium* is an increasing threat to endangered species of birds throughout the world [30–33]. Many captive bird species are susceptible to various malarial infections [7]. Zoos have important role in species conservation, and cases of mortality in different species of birds due to malaria and related haemosporidians were described, particularly often in countries with warm climates all over the planet [9, 10, 13, 15, 16, 18]. Although it is known that the same lineages of malaria parasites can infect birds belonging to different species, families and even orders, there is insufficient knowledge about the prevalence and diversity of *Plasmodium* spp. and relative haemosporidians in entire collections of zoo birds; usually only information about malaria in certain species of birds is published [9, 10, 13, 15, 16, 18]. Here, the first study of distribution of *Plasmodium* spp. and *Haemoproteus* spp. in captive birds belonging to 17 orders (Table 1; Fig. 9) is reported. This corresponds to almost half of all the orders currently accepted in taxonomy of birds.

This study shows high prevalence of *Plasmodium* and *Haemoproteus* parasites (12.6%) in captive birds in the São Paulo Zoo. Although there are no similar studies using such large number of different bird species for comparison, some studies in zoos have been carried out in Brazil, and they show similar prevalence data. In a study with penguins kept under captive conditions in rehabilitation centres in Brazil [17], the overall prevalence of infected birds was similar to that reported here. However, when we compare our results with the data obtained for captive psittacine birds from three zoological gardens from Brazil [9], the prevalence of infection was much lower in this study. This difference may be explained by the adaptation of native Brazilian psittacine birds to malarial infections and resulting increased susceptibility to malaria compared to some non-native species that were analysed in this study. Many of them are not native to Brazil (Table 1; Additional file 1) and, therefore, might be less susceptible to local parasites or less attractive to the vectors of these parasites. On the other hand, these data might reflect specific epidemiology situation at our study site where the prevalence of haemosporidians in free-living birds [21] was quite similar to the overall prevalence reported in zoo collection. However, it is important to note that the majority of samples analysed were from animals that needed some kind of veterinary care. This could have contributed for the prevalence of infection to be higher than it would have been if samples were collected randomly. Moreover, the presence of lethal abortive haemosporidian infections on tissue stage when parasitaemia is absent also cannot be ruled out, and that might explain low prevalence of infection when solely examination of blood samples is carried out [11, 34]. Abortive development of tissue meronts might be underestimated by molecular diagnostics or microscopic examination of blood samples, and histological studies are needed to determine parasites in organs [35–37]. It worth mentioning that the Anatidae birds are particularly susceptible and thus are at risk to acquire haemosporidian parasites in the São Paulo Zoo. These data are in accordance with the former experimental observations indicating high susceptibility of ducks and geese to many species of *Plasmodium* [7]. In the São Paulo Zoo, anatids live close to a big lake surrounded by suitable vegetation and consequently, near to the breeding and resting site of female mosquitoes.

Infections of *Plasmodium* spp. predominated when compared to those of the *Haemoproteus* spp., indicating active involvement of mosquitoes in haemosporidian transmission in the São Paulo Zoo. Species of *Haemoproteus* are transmitted by *Culicoides* biting midges and hippoboscid flies [7, 38]. These parasites seem to be more specific to avian hosts [7, 19], and that probably restricts their transmission between zoo birds belonging to far-distant taxa. However, *Haemoproteus* parasites might cause severe disease and even kill non-adapted birds on tissue stage before development of parasitaemia [12] thus worth more attention in veterinary medicine studies in zoos. That requires investigation of tissue stages of dead birds by application traditional histology and chromogenic in situ hybridization methods [37, 39]. Distribution of biting midges and hippoboscid flies in zoos remains insufficiently investigated.

After consulting the MalAvi database, it was found that three species of birds have been already reported as hosts of haemosporidian parasites: *Alopochen aegyptiaca*, *Psarocolius decumanus* and *Tadorna ferruginea*. Additionally, GenBank contains information about reports of *Plasmodium* and *Haemoproteus* spp. sequences in *Dendrocygna viduata*, *Phoenicopterus chilensis*, *Pipile jacutinga* and *Saltator atricolis*. All the other bird species found infected in this study represent the first records of lineages of haemosporidian parasites.

The lineage pNYCNYC01 was the most prevalent lineage, and it was found in 40% of the positive animals belonging to 16 bird species, indicating that it might be generalist parasite (Table 1; Fig. 9). However, presence of blood stages was not documented in all blood films indicating possible abortive development in some host species, as was the case in a recent study in Ecuador [40]. This lineage was previously reported in the São Paulo Zoo infecting free-living birds, namely *Nycticorax nycticorax* (Pelecaniformes: Ardeidae) and *Penelope superciliaris* (Galliformes: Cracidae) [21]. Because of its high similarity (99%) with the lineage pPESA01, it possible that they are variants of the same parasite species. The lineage pPESA01 of *Plasmodium* spp. was found infecting 4 bird species in this study, and has been reported in different parts of the world: Alaska [41], Uruguay [42] and Brazil [43] in birds of Charadriiformes, Columbiformes and Passeriformes, respectively. Erythrocytic stages of the lineage pNYCNYC01 were seen in the blood films of *Cygnus atratus, Sarcoramphus papa, Tadorna ferruginea, Tadorna variegata, Dendrocygna viduata* and *Anser cygnoides* in this study, and that indicates competent hosts and complete (non-abortive) development. However, blood stages of this parasite lineage were always at light parasitaemia and/or associated with *Haemoproteus* sp., making difficult the convincing morphological identification of *Plasmodium* species. To develop molecular characterization of this and other *Plasmodium* parasites found in this study, it will be helpful to perform experimental infections, during which high parasitaemia usually develop and all blood stages, necessary for parasite identification occur [7, 44]. This is the most efficient way to describe new avian *Plasmodium* species and develop molecular characterization of described ones. Such studies are important, because there is still a remarkable gap in taxonomic and molecular characterization of haemosporidian species in South America [19].

The high prevalence of the pNYCNYC01 lineage in birds and light parasitaemia may indicate that this lineage likely is adapted to Anatidae species and is transmitted in the zoo quite a while. Transmission of this parasite certainly occurs in the São Paulo Zoo because it was reported in 22 Anatidae birds, which were born at the study site, including two juvenile *Cygnus atratus*. In fact, generalist parasites have high ability for dispersion [45, 46]. In this case, the high host diversity contributes to the increase of rate of parasite transmission due to increased number of susceptible vertebrate hosts resulting in high parasite prevalence [47]. Generalist parasites are also predicted to occupy larger geographic ranges because of their capacity to exploit a variety of hosts and environments (niche breadth hypothesis) [48]. Avian parasites can benefit from their vertebrate hosts’ broad geographical distribution [49, 50] and, although the lineage pNYCNYC01 has been reported only in America continent, the lineage pPESA01, a possible variant of pNYCNYC01, has been found even in Alaska birds, but the presence of gametocytes were not documented in Alaska. It is important to note that PCR based records of a lineage in a host species is not enough evidence of the species being a competent host. The latter term implies complete life cycle and presence of infective stages (gametocytes) in the circulation, and that is different from simple presence of parasite DNA. There is increasing evidence that haemosporidian infections in non-competent hosts result in partial (abortive) development of the parasites before they reach the stage of infectious gametocytes. Because such DNA may leak into the blood, it is possible that the host ranges across bird taxa is overestimating the ranges of their competent hosts [34, 36, 40]. Thus, microscopic examination of blood films remains a gold method in field haemosporidian parasite studies.

The megadiverse host environment of São Paulo Zoo, composed by native species from the Atlantic forest, captive species from this and other Brazilian biomes associated to captive exotic species probably gave benefits for generalist parasites in their transmission. In fact, the lineage pNYCNYC01 was the third more generalist lineage of *Plasmodium* described so far; it was reported in birds of 7 orders (Fig. 9), while the *P. relictum* lineages pSGS1 and pGRW04 have been found, according the MalAvi database, in birds of 11 orders. The lineage pGRW04 of *P. relictum* is a widespread and is markedly virulent in many bird species [51]. Among other generalist *Plasmodium* parasites, the lineages pPADOM09, pTURDUS1, pSEIAUR01, pGALLUS01 and pSW5 should be mentioned; these lineages belong to *P. elongatum*, *Plasmodium circumflexum*, *Plasmodium cathemerium*, *Plasmodium gallinaceum* and *P. circumflexum*, respectively. This further reinforces the need to continue research on the pNYCNYC01 and pPESA01 lineages, aiming their both the morphological and molecular characterizations.

It is important to note that *P. relictum* is distributed worldwide in a broad range of vertebrate hosts, and its life cycle has been studied relatively well [7]. This cosmopolitan malaria parasite contains 4 different lineages (pGRW04, pGRW11, pLZFUS01 and pSGS1) [52–55]. Interestingly, *P. relictum* has been reported rarely in Brazil [6] and also it was not detected during this study. This is probably due to a geographical isolation and absence of introduction of this infection rather than absence of competent vectors or susceptible avian host, because (1) *Culex quinquefasciatus* mosquitoes, the main vectors of *P. relictum* (pGRW4), is common in São Paulo [56] and (2) susceptible to *P. relictum* avian host species present in the zoo [7]. Because *P. relictum* (pGRW04, pSGS1) is markedly virulent in non-adapted hosts and even can cause mortality [51, 55], we call for strict veterinary control when importing birds from other parts of the world because that might lead to introduction of new malaria infections to Brazil.

The lineage pDENPET03 of *P. nucleophilum*, the second most common lineage in this study, was found infecting mainly Anatidae species. The previously developed molecular characterization of this infection [10] was essential during this study because blood stages were not visible in blood films of the majority of PCR positive bird samples. This parasite lineage has been reported in South and North America in birds of Anseriformes [10], Charadriiformes [57], Passeriformes [6, 42, 43, 58–62], Psittaciformes [42] and Sphenisciformes [63]. However, presence of gametocytes of *P. nucleophilum* has been documented only in birds of the Anatidae [10], and it remains unclear if this parasite completes life cycle in all mentioned birds.

The lineage pMYCAME02 was found in two hosts at our study site that belong to two different orders (Anseriformes and Phoenicopteriformes). This lineage has been formerly reported in North and Midwest Brazil infecting *Mycteria americana* (Ciconiiformes: Ciconiidae) [64] and in Alaska infecting *Dendroica steophaga* (Passeriformes: Parulidae) [62].

The pARACAJ01 lineage belongs to *Plasmodium* sp. and was found in *Aramides cajaneus* (Rallidae). This is a new lineage, which is of 96% similarity to the lineage pLEPCOR04 described in a Passeriformes bird (*Lepidothrix coronata*) in South America [65]. It is probable that the lineage pARACAJ01 belong to undescribed *Plasmodium* species because the most similar lineage (95% of similarity) belong to *Plasmodium unalis* (pTFUS06); the genetic difference of 5% in *cytb* gene is high between these lineages, indication their possible different species status [34]. The lineage pNOTURU01 was reported in *Nothocrax urumutum* (Cracidae). Although we did not have positive blood smears from this sample, this malaria parasite probably belongs to *Haemamoeba* subgenus, since it clustered along with species of this subgenus (Fig. 4). This lineage is most similar (of 98% similarity) to the lineage pCINCHA01 that was found in different Passeriformes species in Africa [66, 67].

The lineage pPADOM09 has been formerly attributed to *P. (Huffia) elongatum* [68], but it clustered together with *P. (Haemamoeba) cathemerium* and not with other *P. elongatum* lineages in our phylogenetic analysis. Because *P. elongatum* is a generalist malaria parasite and the lineage pPADOM09 was not widely distributed in the zoo, it seems that the attribution of pPADOM09 to *P. elongatum* likely was incorrect, as has been mentioned in several studies [19, 43, 69]. The lineage pPADOM09 was recorded only in one individual of *Anser cygnoides* that lived with other Anatidae species in a huge zoo lake.

The lineage pSPMAG06 of *Plasmodium* sp. was found in *Musophaga violacea*. It is of 99% similarity with the lineage pTFUS05 of *Plasmodium lutzi* reported in *Turdus fuscater* (Great Thrush) from Colombia [70]. It is likely that the lineage pSPMAG06 belong to this species, although that could not to be confirmed morphologically because blood stages were not seen in blood smears. During this study, the lineage pSPMAG06 was detected only one time, but it was already reported in the São Paulo Zoo seven years ago [16]. Its low prevalence could be due to the lack of specific vector or susceptible vertebrate hosts in the area, but even so, it might be harmful to some animal that can be introduced in the zoo in the future, especially penguins [17, 63].

The lineage pSALAT01 of *Plasmodium* sp. is new. It was found in *Saltator atricolis* (Thraupidae). In the blood smears, we detected parasites, which have morphological features characteristic of different *Plasmodium* subgenera, but only one sequence was obtained. This sequence was of 97% similarity with the *Plasmodium* sp. lineage pTUMIG03 found in other species of Passeriformes [42, 43, 58, 62, 71–74] and Sphenisciformes [63].

The lineage pMITOM01 of *Plasmodium* sp. was found in *Mitu tomentosum* (Cracidae) and *Phoenicopterus chilensis* (Phoenicopteridae). It was of 98% similarity with the lineage pVIOLI07, which was reported in *Vireo olivaceus,* a migrant species of Passeriformes examined in Colombia [75].

Two new lineages *Haemoproteus* spp. were determined during this study. The lineage hPENOBS01 was detected in *Penelope obscura* (Galliformes: Cracidae); it is most similar to the lineage hMILANS03, with 96% similarity between their partial *cytb* sequences. However, the lineage hMILANS03 was discovered in Spain infecting *Milvus migrans* (Accipitriformes: Accipitridae) [76]. Experimental studies show that same *Haemoproteus* spp. usually do not complete life cycle in birds belonging to different orders [7]. Moreover, the lineages hPENOBS01 and hMILANS03 appeared in different clades in phylogenetic tree (Fig. 5), indicating that they likely belong to different *Haemoproteus* species. Several species of *Haemoproteus* have been described infecting Galliformes birds [7]. This study showed that the lineage hPENOBS01 belong to *H. ortalidum,* the parasite of Galliformes birds described in Venezuela [77]. This *Haemoproteus* species has both elongate and roundish gametocytes, possessing large and clear circular vacuoles in macrogametocytes, as visualised in hPENOBS01 parasites found in blood smears (Fig. 7). The *H. ortalidum cytb* sequence is now established, and it can be used for molecular identification (barcoding) of this infection. It is important to note that *H. ortalidum* is likely transmitted by *Culicoides* biting midges based on our phylogenetic analysis (Fig. 5) because its DNA sequence cluster with parasites of subgenus *Parahaemoproteus*, all investigated species of which are transmitted by these blood-sucking insects [78].

The *Haemoproteus* lineage hEUDRUB01 had 99% of similarity with the lineage hHALMAL01 that was found in *Halcyon malimbica* in Africa [69]. Hosts of these parasite lineages belong to different orders, i.e. (Pelecaniformes and Coraciiformes, respectively). It also was of 99% similarity with the lineage hFREAND01 of *Haemoproteus valkiunasi*, which parasitize *Fregata andrewsi*, *F. minor* and *F. magnificens* (Pelecaniformes). Other *Haemoproteus* spp. of close genetic similarity (98%) to hEUDRUB01 are the lineage hCIRCUM01 of *Haemoproteus noctuae* (the parasite of Strigiformes birds), the lineage hPICAN02 of *Haemoproteus homovelans* (the parasite of Piciformes) and the lineage hALCLEU01 of *Haemoproteus enucleator* (the parasite of Coraciiformes). Although gametocytaemia of the lineage hEUDRUB01 was light in our samples, it is likely that parasite of this lineage do not belong to any of the mentioned *Haemoproteus* species because it was reported in birds of different orders and gametocytes differ morphologically.

It is important to mention that although some lineages have been found in single or few species of birds in this study, and such lineages look to be specialists this could be an effect of the sampling bias because a few individual birds were sampled for some host species (Table 1; Additional file 1). These parasites could eventually be found in more bird species if sampling could be expanded and more bird species would be involved in investigation.

It worth mentioning that *Leucocytozoon* spp. was not found in blood films of all examined birds, indicating a possible absence of transmission of these haemosporidian parasites in the zoo. In fact, *Leucocytozoon* spp. have not been detected in Brazil [7] but the reason is still unclear, although there are far fewer studies that have tested for *Leucocytozoon* spp. than for either *Plasmodium* or *Haemoproteus* spp. [19].

Seasonal variation in the prevalence of vector-borne diseases is well documented [79]. Although not statistically significant, a higher infection prevalence was found in summer than in the other seasons, a pattern that has been observed in other studies of avian malaria in Brazil [63]. To verify if climatic factors may have influenced our analysis, the average precipitation and minimum average temperature data recorded in the period of the study were examined. In fact, there was an unusual year in precipitation levels, but this did not change significantly the combined data of the period of the study (Additional file 2). However, the management activities performed in a big central lake of the zoo, in which very large number of animals was collected in a short period of time, mainly in the autumn, may have affected the analysis.

All samples with positive blood smears were also PCR positive, but otherwise did not occurred probably showing that, in captive conditions where animals do not have to compete for food or shelter, parasitaemia might be light and molecular techniques can be more sensitive for parasites detection than microscopy. Once the individuals have good body condition and receive veterinary care, surviving of acute stage of infection could be easier, increasing the survival chances and the number of light chronic infections. However, these results also might be due to possible abortive development of some infections when they appear in non-adapted hosts as is the case in captive parrots in Europe [11, 12]. In the latter case only tissue stages develop, their merozoites or remnants of tissue stages (syncytia) appear in circulation providing templates for PCR amplification, but parasite cannot inhabit red blood cells and thus are difficult to detect by microscopic examination of blood films [36, 40]. Such haemosporidian infections might be virulent, but remains insufficiently investigated. Haemosporidian infections have been insufficiently investigated in captive animals, and few studies have been addressed this issue [10, 11, 13, 15, 16, 63]. Birds often live longer in captivity than in wildlife, and light chronic infections usually are predominant. Understanding the role of such infections is essential to guarantee quality of bird life, and preserve their reproduction and longevity. Chronically infected birds could have their reproductive fitness affected, producing fewer eggs and a compromised offspring [80, 81]. This is an important concern when dealing with animals that are involved in conservation programmes. It is important to note that testing of birds for haemoparasites by PCR-based methods is important before sending to or receiving birds from other zoos. Nowadays, this is not always taken into account, but the introduction of new infections in environments were transmission possible might lead to devastating consequences, as is the case in Hawaii where endemic birds are dying of avian malaria and there is no good solutions how to stop distribution of this disease [31, 51]. Moving animals between different enclosures is common in zoos, and parasite positive animals should be maintained in mosquito-free facilities during treatment. Zoos usually occupy big sites, and even transfer of an infected bird from its original enclosure to another one (for example, to receive treatment from the veterinary staff) could be enough to infect susceptible mosquitoes, which can establish parasite transmission to other hosts. Treatment of haemosporidian infections is also important to prevent chronic infections and to decrease of transmission along the years, but remains insufficiently developed in avian haemosporidian parasites because of lack of effective drugs, which are effective against tissue stages of the parasites [7, 82].

Environmental conditions have a strong influence in haemosporidian vector distribution. *Plasmodium* parasites slow down sporogonic development in vectors when temperature is below 15 °C. Available data show that the transmission decreases during the autumn and winter periods in the Holarctic [7, 83]. Besides low temperature, these seasons often are characterized by low precipitation in tropical regions, and that influences vector population that depends on water availability necessary for their breeding. In this study, some seasonality of transmission was seen, since the most of cases were detected shortly after the end of summer, when rainfall rates begin to decrease. However, that also might be related to seasonal relapses of infections, and that remains insufficiently studied in tropical countries [7]. Although it is important to establish strategies of pest management in the São Paulo Zoo that requires a well-developed programme of control aiming prevent possible harm to zoo animals. Some success was reported in vector control in zoos, in which fish (*Gambusia* sp.) and larvicides based on *Bacillus thuringiensis* var. *israelensis* (Bti) or *Bacillus sphaericus* were used in water bodies [13, 84]. Additional studies are needed to develop best strategies aiming to decrease densities of mosquitoes and other vectors and to reduce the prevalence of avian haemosporidians in zoos.

It worth mentioning that survival of acute primary parasitaemia and subsequent development of light chronic parasitaemia are not always indications of improved health during avian malaria because of possible development of secondary exoerythrocytic meronts, which often kill birds [37]. That requites permanent control of chronically infected birds in zoos and calls for development of measures to treat completely such infections.

## Conclusion

Zoos have an important role in animal species conservation, but the threat of parasites and parasitic infections is constantly present. This study showed that many bird species are at risk to acquire malaria in São Paulo Zoo. Thus, it is important to develop and keep quarantine protocols for animals that are going to be incorporated in the bird collection as well as for those going to be sent to other institutions. That is essential to avoid or minimize the introduction of parasites in new sites where transmission is possible, but still absent due to absence of agents of infections. Because it is difficult or even impossible to prevent contact between free-living and captive birds in zoos located in forest remnants, the creation of preventive protocols, such as periodic examinations, quarantine isolation and treatment of infected animals, are essential. Occurrence of abortive haemosporidian infections, which might kill birds before development of parasitaemia, remains insufficiently understood in zoos and needs additional research. Both microscopic and PCR-based examination of blood samples are often insufficient methods to detect abortive haemosporidian infections, which damage internal organs. Application of histology and chromogenic in situ hybridization methods worth more broad application in zoological gardens for better understanding avian pathology and diseases caused by haemosporidian parasites.

## Additional files

**Additional file 1.** Birds with negative results of PCR-based diagnostics of *Plasmodium* and *Haemoproteus* parasites. The data provided represent the birds with negative results for *Plasmodium* and *Haemoproteus* parasites, obtained by PCR.

**Additional file 2.** Average precipitation and minimum average temperature data recorded in the period of the study. The data provided represent the averages of precipitation and minimum temperature registered during the period of this study.

## References

[1] Olney PJ (2005). Building a future for wildlife: the world zoo and aquarium conservation strategy.

[2] EAZA. The Modern Zoo: Foundations for Management and Development. EAZA Executive Office. Amsterdam, the Netherlands. 2nd Edition; 2013.

[3] Mukhin A, Palinauskas V, Platonova E, Kobylkov D, Vakoliuk I, Valkiūnas G (2016). The strategy to survive primary malaria infection: an experimental study on behavioural changes in parasitized birds. PLoS ONE.

[4] Atkinson CT, Thomas NJ, Hunter DB (2008). Parasitic diseases of wild birds.

[5] Panayotova-Pencheva MS (2013). Parasites in captive animals: a review of studies in some European zoos. Zool Garten..

[6] Marzal A, Ricklefs RE, Valkiūnas G, Albayrak T, Arriero E, Bonneaud C (2011). Diversity, loss, and gain of malaria parasites in a globally invasive bird. PLoS ONE.

[7] Valkiūnas G (2005). Avian malaria parasites and other Haemosporidia.

[8] Murata K, Nii R, Sasaki E, Ishikawa S, Sato Y, Sawabe K (2008). *Plasmodium* (Bennettinia) *juxtanucleare* infection in a captive white eared-pheasant (*Crossoptilon crossoptilon*) at a Japanese zoo. J Vet Med Sci.

[9] Belo NO, Passos LF, Júnior LM, Goulart CE, Sherlock TM, Braga EM (2009). Avian malaria in captive psittacine birds: detection by microscopy and 18S rRNA gene amplification. Prev Vet Med..

[10] Chagas CRF, Valkiūnas G, Nery CVC, Henrique PC, Gonzalez IHL, Monteiro EF (2013). *Plasmodium* (Novyella) *nucleophilum* from an Egyptian Goose in São Paulo Zoo, Brazil: microscopic confirmation and molecular characterization. Int J Parasitol Parasites Wildl..

[11] Olias P, Wegelin M, Zenker W, Freter S, Gruber AD, Klopfleisch R (2011). Avian malaria deaths in parrots, Europe. Emerg Infect Dis.

[12] Palinauskas V, Iezhova TA, Križanauskienė A, Markovets MY, Bensch S, Valkiūnas G (2013). Molecular characterization and distribution of *Haemoproteus minutus* (Haemosporida, Haemoproteidae): a pathogenic avian parasite. Parasitol Int.

[13] Thurber MI, Gamble KC, Krebs B, Goldberg TL (2014). Molecular detection of *Plasmodium* in free-ranging birds and captive flamingos (*Phoenicopterus chilensis*) in Chicago. J Zoo Wildl Med..

[14] Ferrell ST, Snowden K, Marlar AB, Garner M, Lung NP (2007). Fatal hemoprotozoal infections in multiple avian species in a zoological park. J Zoo Wildl Med..

[15] Scaglione FE, Canizoo FT, Chiappino L, Sereno A, Ripepi M, Salamida S (2016). *Plasmodium* spp. in a captive raptor collection of a Safari park in northwest Italy. Res Vet Sci.

[16] Bueno MG, Lopez RPG, Menezes RMT, Costa-Nascimento MJ, Lima GFMC, Araújo RAS (2010). Identification of *Plasmodium relictum* causing mortality in penguins (*Spheniscus magellanicus*) from São Paulo Zoo. Brazil. Vet Parasitol.

[17] Vanstreels RE, Kolesnikovas CK, Sandri S, Silveira P, Belo NO, Ferreira Junior FC (2014). Outbreak of avian malaria associated to multiple species of *Plasmodium* in magellanic penguins undergoing rehabilitation in southern Brazil. PLoS ONE.

[18] Grilo ML, Vanstreels RE, Wallace R, García-Párraga D, Braga ÉM, Chitty J (2016). Malaria in penguins: current perceptions. Avian Pathol..

[19] Clark NJ, Clegg SM, Lima MR (2014). A review of global diversity in avian haemosporidians (*Plasmodium* and *Haemoproteus*: Haemosporida): new insights from molecular data. Int J Parasitol.

[20] Fundação Parque Zoológico de São Paulo. http://www.zoologico.com.br/wp-content/uploads/2013/07/plantel_zoologico_2015-1.pdf. Accessed 19 Jul 2016.

[21] Chagas CR, Guimarães Lde O, Monteiro EF, Valkiūnas G, Katayama MV, Santos SV (2016). Hemosporidian parasites of free-living birds in the São Paulo Zoo, Brazil. Parasitol Res.

[22] Godfrey RD, Fedynich AM, Pence DB (1987). Quantification of hematozoa in blood smears. J Wildl Dis.

[23] Hellgren O, Waldenström J, Bensch S (2004). A new PCR assay for simultaneous studies of *Leucocytozoon*, *Plasmodium*, and *Haemoproteus* from avian blood. J Parasitol.

[24] Bensch S, Hellgren O, Pérez-Tris J (2009). MalAvi: a public database of malaria parasites and related haemosporidians in avian hosts based on mitochondrial cytochrome b lineages. Mol Ecol Resour..

[25] Huelsenbeck JP, Ronquist F (2001). MRBAYES: Bayesian inference of phylogenetic trees. Bioinformatics.

[26] Rambaut A. FigTree: Tree Figure Drawing Tool Version 1.4.0 2006–2012, Institute of Evolutionary Biology, University of Edinburgh. http://tree.bio.ed.ac.uk/software/figtree.

[27] Bandelt H-J, Forster P, Röhl A (1999). Median-joining networks for inferring intraspecific phylogenies. Mol Biol Evol.

[28] Meteorological Institute of Astronomy, Geophysics and Atmospheric Sciences, São Paulo University. http://www.estacao.iag.usp.br. Accessed 17 Aug 2016.

[29] International Union for Conservation of Nature and National Resources. The IUCN red list of threatened species. Version 2016-2. http://www.iucnredlist.org. Accessed 14 Oct 2016.

[30] Motta RO, Romero Marques MV, Ferreira Junior FC, Andery Dde A, Horta RS, Peixoto RB (2013). Does haemosporidian infection affect hematological and biochemical profiles of the endangered Black-fronted piping-guan (*Aburria jacutinga*)?. PeerJ.

[31] Atkinson CT, Utzurrum RB, Lapointe DA, Camp RJ, Crampton LH, Foster JT (2014). Changing climate and the altitudinal range of avian malaria in the Hawaiian Islands - an ongoing conservation crisis on the island of Kaua’i. Glob Chang Biol..

[32] Schoener ER, Banda M, Howe L, Castro IC, Alley MR (2014). Avian malaria in New Zealand. N Z Vet J..

[33] Neto JM, Pérez-Rodríguez A, Haase M, Flade M, Bensch S (2015). Prevalence and diversity of *Plasmodium* and *Haemoproteus* parasites in the globally-threatened Aquatic Warbler *Acrocephalus paludicola*. Parasitology.

[34] Valkiūnas G, Palinauskas V, Ilgūnas M, Bukauskaitė D, Dimitrov D, Bernotienė R (2014). Molecular characterization of five widespread avian haemosporidian parasites (Haemosporida), with perspectives on the PCR-based detection of haemosporidians in wildlife. Parasitol Res.

[35] Dinhopl N, Mostegl MM, Richter B, Nedorost N, Maderner A, Fragner K (2011). Application of in situ hybridization for the detection and identification of avian malaria parasites in paraffin wax-embedded tissues from captive penguins. Avian Pathol..

[36] Palinauskas V, Žiegytė R, Iezhova TA, Ilgūnas M, Bernotienė R, Valkiūnas G (2016). Description, molecular characterisation, diagnostics and life cycle of *Plasmodium elongatum* (lineage pERIRUB01), the virulent avian malaria parasite. Int J Parasitol.

[37] Ilgūnas M, Bukauskaitė D, Palinauskas V, Iezhova TA, Dinhopl N, Nedorost N (2016). Mortality and pathology in birds due to *Plasmodium* (Giovannolaia) *homocircumflexum* infection, with emphasis on the exoerythrocytic development of avian malaria parasites. Malar J..

[38] Bukauskaitė D, Bernotienė R, Iezhova TA, Valkiūnas G (2016). Mechanisms of mortality in *Culicoides* biting midges due to *Haemoproteus* infection. Parasitology.

[39] Dinhopl N, Nedorost N, Mostegl MM, Weissenbacher-Lang C, Weissenböck H (2015). *In situ* hybridization and sequence analysis reveal an association of *Plasmodium* spp. with mortalities in wild passerine birds in Austria. Parasitol Res.

[40] Moens MA, Valkiūnas G, Paca A, Bonaccorso E, Aguirre N, Pérez-Tris J (2016). Parasite specialization in a unique habitat: hummingbirds as reservoirs of generalist blood parasites of Andean birds. J Anim Ecol.

[41] Yohannes E, Križanauskiené A, Valcu M, Bensch S, Kempenaers B (2009). Prevalence of malaria and related haemosporidian parasites in two shorebird species with different winter habitat distributions. J Ornithol.

[42] Durrant KL, Beadell JS, Ishtiaq F, Graves GR, Olson SL, Gering E (2006). Avian hematozoa in South America: a comparison of temperate and tropical zones. Ornithol Monogr..

[43] Lacorte GA, Félix GM, Pinheiro RR, Chaves AV, Almeida-Neto G, Neves FS (2013). Exploring the diversity and distribution of neotropical avian malaria parasites: a molecular survey from Southeast Brazil. PLoS ONE.

[44] Dimitrov D, Palinauskas V, Iezhova TA, Bernotienė R, Ilgūnas M, Bukauskaitė D (2015). *Plasmodium* spp.: an experimental study on vertebrate host susceptibility to avian malaria. Exp Parasitol.

[45] Clayton DH, Al-Tamimi S, Johnson KP, Page RDM (2003). The ecological basis of co-evolutionary history. Tangled trees: phylogeny, co-speciation, and coevolution.

[46] MacLeod CJ, Paterson AM, Tompkins DM, Duncan RP (2010). Parasites lost: do invaders miss the boat or drown on arrival?. Ecol Lett.

[47] Keesing F, Holt RD, Ostfeld RS (2006). Effects of species diversity on disease risk. Ecol Lett.

[48] Krasnov BR, Poulin R, Shenbrot GI, Mouillot D, Khokhlova IS (2005). Host specificity and geographic range in haematophagous ectoparasites. Oikos.

[49] Pérez-Tris J, Bensch S (2005). Diagnosing genetically diverse avian malarial infections using mixed-sequence analysis and TA-cloning. Parasitology.

[50] Hellgren O, Waldenström J, Peréz-Tris J, Szöll E, Si O, Hasselquist D (2007). Detecting shifts of transmission areas in avian blood parasites: a phylogenetic approach. Mol Ecol.

[51] van Riper C (1991). The impact of introduced vectors and avian malaria on insular passeriform bird population in Hawaii. Bull. Soc. Vector Ecol..

[52] Ilgūnas M, Palinauskas V, Iezhova TA, Valkiūnas G (2013). Molecular and morphological characterization of two avian malaria parasites (Haemosporida: Plasmodiidae), with description of *Plasmodium homonucleophilum* n. sp. Zootaxa..

[53] Palinauskas V, Kosarev V, Shapoval A, Bensch S, Valkiūnas G (2007). Comparison of mitochondrial cytochrome b lineages and morphospecies of two avian malaria parasites of the subgenera Haemamoeba and Giovannolaia (Haemosporida: Plasmodiidae). Zootaxa..

[54] Valkiūnas G, Zehtindjiev P, Hellgren O, Ilieva M, Iezhova TA, Bensch S (2007). Linkage between mitochondrial cytochrome b lineages and morphospecies of two avian malaria parasites, with a description of *Plasmodium* (Novyella) *ashfordi* sp. nov. Parasitol Res.

[55] Palinauskas V, Valkiūnas G, Bolshakov CV, Bensch S (2008). *Plasmodium relictum* (lineage P-SGS1): effects on experimentally infected passerine birds. Exp Parasitol.

[56] Ceretti-Junior W, de Oliveira Christe R, Rizzo M, Strobel RC, de Matos Junior MO, de Mello MH, Fernandes A, Medeiros-Sousa AR (2015). Species composition and ecological aspects of immature mosquitoes (Diptera: Culicidae) in Bromeliads in Urban Parks in the City of São Paulo. Brazil. J Arthropod Borne Dis..

[57] Roos FL, Belo NO, Silveira P, Braga EM (2015). Prevalence and diversity of avian malaria parasites in migratory Black Skimmers (Rynchops niger, Laridae, Charadriiformes) from the Brazilian Amazon Basin. Parasitol Res.

[58] Ricklefs RE, Fallon SM (2002). Diversification and host switching in avian malaria parasites. Proc Biol Sci..

[59] Szymanski MM, Lovette IJ (2005). High lineage diversity and host sharing of malarial parasites in a local avian assemblage. J Parasitol.

[60] Pagenknopp KM, Klicka J, Durrant KL, Garvin JC, Fleischer RC (2008). Geographic variation in malaria parasite lineages in the common yellowthroat (*Geothlypis trichas*). Conserv Genetics..

[61] Levin II, Zwiers P, Deem SL, Geest EA, Higashiguchi JM, Iezhova TA (2013). Multiple lineages of avian malaria parasites (*Plasmodium*) in the Galapagos Islands and evidence for arrival via migratory birds. Conserv Biol.

[62] Oakgrove KS, Harrigan RJ, Louiseau C, Guers S, Seppi B, Sehgal RN (2014). Distribution, diversity and drivers of blood-borne parasite co-infections in Alaskan bird populations. Int J Parasitol.

[63] Vanstreels RE, da Silva-Filho RP, Kolesnikovas CK, Bhering RC, Ruoppolo V, Epiphanio S (2015). Epidemiology and pathology of avian malaria in penguins undergoing rehabilitation in Brazil. Vet Res.

[64] Villar CM, Bryan AL, Lance SL, Braga EM, Congrains C, Del Lama SN (2013). Blood parasites in nestlings of wood stork populations from three regions of the American continent. J Parasitol.

[65] Moens MA, Pérez-Tris J (2016). Discovering potential sources of emerging pathogens: South America is a reservoir of generalist avian blood parasites. Int J Parasitol.

[66] Lauron EJ, Loiseau C, Bowie RC, Spicer GS, Smith TB, Melo M (2015). Coevolutionary patterns and diversification of avian malaria parasites in African sunbirds (Family Nectariniidae). Parasitology.

[67] Loiseau C, Harrigan RJ, Robert A, Bowie RC, Thomassen HA, Smith TB (2012). Host and habitat specialization of avian malaria in Africa. Mol Ecol.

[68] Escalante AA, Freeland DE, Collins WE, Lal AA (1998). The evolution of primate malaria parasites based on the gene encoding cytochrome b from the linear mitochondrial genome. Proc Natl Acad Sci USA.

[69] Beadell JS, Covas R, Gebhard C, Ishtiaq F, Melo M, Schmidt BK (2009). Host associations and evolutionary relationships of avian blood parasites from West Africa. Int J Parasitol.

[70] Mantilla JS, Matta NE, Pacheco MA, Escalante AA, González AD, Moncada LI (2013). Identification of *Plasmodium* (Haemamoeba) *lutzi* (Lucena, 1939) from *Turdus fuscater* (Great Thrush) in Colombia. J Parasitol.

[71] Martinsen ES, Waite JL, Schall JJ (2007). Morphologically defined subgenera of *Plasmodium* from avian hosts: test of monophyly by phylogenetic analysis of two mitochondrial genes. Parasitology.

[72] Merino S, Moreno J, Vásquez RA, Martínez J, Sánchez-Monsálvez I, Estades CF (2008). Haematozoa in forest birds from southern Chile: latitudinal gradients in prevalence and parasite lineage richness. Austral Ecol.

[73] Dodge M, Guers SL, Sekercioğlu ÇH, Sehgal RN (2013). North American transmission of hemosporidian parasites in the Swainson’s thrush (*Catharus ustulatus*), a migratory songbird. J Parasitol.

[74] Martínez J, Vásquez RA, Venegas C, Merino S (2015). Molecular characterisation of haemoparasites in forest birds from Robinson Crusoe Island: is the Austral Thrush a potential threat to endemic birds. Bird Conserv Int..

[75] González AD, Lotta IA, García LF, Moncada LI, Matta NE (2015). Avian haemosporidians from Neotropical highlands: evidence from morphological and molecular data. Parasitol Int.

[76] Pérez-Rodríguez A, de la Puente J, Onrubia A, Pérez-Tris J (2013). Molecular characterization of haemosporidian parasites from kites of the genus *Milvus* (Aves: Accipitridae). Int J Parasitol.

[77] Gabaldon A, Ulloa G (1978). Subespecie de *Haemoproteus rotundus* Oliger, 1956 (Haemosporina: Haemoproteidae) presente en Venezuela. Boletin de la Direccion de Malariologia y Saneamiento Ambiental..

[78] Bukauskaitė D, Žiegytė R, Palinauskas V, Iezhova TA, Dimitrov D, Ilgūnas M (2015). Biting midges (Culicoides, Diptera) transmit *Haemoproteus* parasites of owls: evidence from sporogony and molecular phylogeny. Parasit Vectors..

[79] Lapointe DA, Atkinson CT, Samuel MD (2012). Ecology and conservation biology of avian malaria. Ann N Y Acad Sci.

[80] Knowles SC, Palinauskas V, Sheldon BC (2010). Chronic malaria infections increase family inequalities and reduce parental fitness: experimental evidence from a wild bird population. J Evol Biol.

[81] Asghar M, Hasselquist D, Hansson B, Zehtindjiev P, Westerdahl H, Bensch S (2015). Chronic infection. Hidden costs of infection: chronic malaria accelerates telomere degradation and senescence in wild birds. Science.

[82] Palinauskas V, Valkiūnas G, Krizanauskiene A, Bensch S, Bolshakov CV (2009). *Plasmodium relictum* (lineage P-SGS1): further observation of effects on experimentally infected passeriform birds, with remarks on treatment with Malarone. Exp Parasitol.

[83] Santiago-Alarcon D, Palinauskas V, Schaefer HM (2012). Diptera vectors of avian Haemosporidian parasites: untangling parasite life cycles and their taxonomy. Biol Rev Camb Philos Soc.

[84] Tuten HC (2011). Habitat characteristics of larval mosquitoes in zoos of South Carolina, USA. J Am Mosq Control Assoc..

