# Supplementary material for: Diversity and distribution of avian malaria and related haemosporidian parasites in captive birds from a Brazilian megalopolis
Source: Malar J. 2017 Feb 17;16:83. doi: 10.1186/s12936-017-1729-8 (PMC5316177; doi:10.1186/s12936-017-1729-8)
Supplement: Supplementary file 1 — Additional file 1. Birds with negative results of PCR-based diagnostics of Plasmodium and Haemoproteus parasites. The data provided represent the birds with negative results for Plasmodium and Haemoproteus parasites, obtained by PCR. [file 12936_2017_1729_MOESM1_ESM.docx]

Additional file 1

Birds with negative results of PCR-based diagnostics of *Plasmodium* and *Haemoproteus* parasites

| ORDER  Family | *Host species*  (Common name) | BIRDS  (SAMPLES) | GEOGRAPHIC RANGE  (Countries of native occurrence) | IUCN STATUS |
| --- | --- | --- | --- | --- |
| ACCIPITRIFORMES |  |  |  |  |
| Accipitridae | *Buteogallus coronatus*  (Crowned Eagle) | 2 (6) | Brazil, Bolivia, Paraguay, Argentina | EN |
|  | *Buteogallus lacernulatus*  (White-necked Hawk) | 4 (5) | Brazil | VU |
|  | *Buteogallus meridionalis*  (Savanna Hawk) | 2 (6) | South and Central America | LC |
|  | *Geranoaetus albicaudatus*  (White-tailed Hawk) | 2 (2) | South and Central America | LC |
|  | *Geranoaetus melanoleucus*  (Black-chested Buzzard-Eagle) | 2 (2) | South America | LC |
|  | *Haliaeetus vocifer*  (African Fish-Eagle) ∆ | 1 (3) | Africa | LC |
|  | *Harpia harpyja*  (Harpy Eagle) | 7 (13) | South and Central America | NT |
|  | *Leptodon cayanensis*  (Gray-headed Kite) | 1 (1) | South and Central America | LC |
|  | *Pseudastur polionotus*  (Mantled Hawk) | 1 (1) | Brazil, Uruguay, Paraguay | NT |
|  | *Spizaetus ornatus*  (Ornate Hawk-Eagle) | 10 (15) | South and Central America | NT |
|  | *Spizaetus tyrannus*  (Black Hawk-Eagle) | 6 (10) | South and Central America * | LC |
|  | *Trigonoceps occipitalis*  (White-headed Vulture) ∆ | 1 (5) | Africa | CR |
| Cathartidae | *Vultur gryphus*  (Vulturine Guineafowl) | 1 (1) | Venezuela, Colombia, Ecuador, Peru, Bolivia, Paraguay, Argentina and Chile | NT |
| ANSERIFORMES |  |  |  |  |
| Anatidae | *Branta canadensis*  (Canada Goose) ∆ | 1 (1) | North America | LC |
|  | *Chenonetta jubata*  (Maned Duck) ∆ | 1 (7) | Oceania | LC |
|  | *Dendrocygna bicolor*  (Fulvous-whistling Duck) | 11 (17) | Africa, South and Central America | LC |
|  | *Tadorna radjah*  (Radjah Shelduck) ∆ | 2 (5) | Oceania | LC |
|  | *Tadorna tadornoides*  (Australian Shelduck) ∆ | 2 (2) | Oceania | LC |
| Anhimidae | *Chauna torquata*  (Southern Screamer) | 1 (2) | South America | LC |
| BUCEROTIFORMES |  |  |  |  |
| Bucerotidae | *Buceros rhinoceros*  (Rhinoceros Hornbill) ∆ | 1 (5) | Thailand, Malaysia, Indonesia, Brunei | NT |
| Bucorvidae | *Bucorvus abyssinicus*  (Abyssinian-ground Hornbill) ∆ | 4 (10) | Africa | LC |
| CARIAMIFORMES |  |  |  |  |
| Cariamidae | *Cariama cristata*  (Red-legged Seriema) | 2 (7) | South America | LC |
|  | *Chunga burmeisteri*  (Black-legged Seriema) | 3 (14) | South America | LC |
| CASUARIIFORMES |  |  |  |  |
| Casuariidae | *Dromaius novaehollandiae* (emu) ∆ | 6 (14) | Australia | LC |
| CUCULIFORMES |  |  |  |  |
| Musophagidae | *Tauraco leucotis*  (White-cheeked Turaco) ∆ | 4 (8) | Sudan, Ethiopia | LC |
| FALCONIFORMES |  |  |  |  |
| Falconidae | *Milvago chimachima*  (Yellow-headed Caracara) | 1 (1) | South and Central America | LC |
|  | *Falco femoralis*  (Aplomado Falcon) | 1 (3) | South and Central America | LC |
| GALLIFORMES |  |  |  |  |
| Cracidae | *Crax blumenbachii*  (Red billed Curassow) | 1 (3) | Brazil | EM |
|  | *Crax rubra rubra*  (Great Curassow) | 1 (1) | Central America, North of South America | VU |
|  | *Mitu tuberosum*  (Razor-billed Curassow) | 1 (1) | South America | LC |
| Odonthophoridae | *Odontophorus capueira*  (Spot-winged Wood-quail) | 3 (3) | Argentina, Brazil, Paraguay | LC |
| GRUIFORMES |  |  |  |  |
| Gruidae | *Balearica regulorum*  (Gray Crowned-Crane) ∆ | 6 (15) | Africa | EM |
| PASSERIFORMES |  |  |  |  |
| Cotingidae | *Pyroderus scutatus*  (Red-ruffed Fruitcrow) | 1 (1) | South America | LC |
|  | *Rupicola rupicola*  (Guianan Cock-of-the-rock) | 1 (6) | North of South America | LC |
| Sturnidae | *Acridotheres cristatellus*  (Crested Myna) ∆ | 2 (2) | Asia | LC |
| PELECANIFORMES |  |  |  |  |
| Pelecanidae | *Pelecanus onocrotalus*  (Great White Pelican) ∆ | 3 (4) | Africa, Middle East | LC |
| Threskionithidae | *Platalea ajaja*  (Roseate Spoonbill) | 2 (3) | South and Central America | LC |
|  | *Theristicus caudatus*  (Buff-necked Ibis) | 1 (1) | South and Central America | LC |
| PHOENICOPTERIFORMES |  |  |  |  |
| Phoenicopteridae | *Phoeniconaias minor*  (Lesser Flamingo) ∆ | 9 (12) | Kenya, Tanzania, Ethiopia, India, Pakistan | NT |
|  | *Phoenicopterus ruber*  (American Flamingo) ∆ | 6 (6) | Bolivia, Venezuela, Central and North America | LC |
| PICIFORMES |  |  |  |  |
| Ramphastidae | *Pteroglossus aracari*  (Black-necked Aracari) | 4 (11) | North of South America | LC |
|  | *Pteroglossus bailloni*  (Saffron Toucanet) | 3 (7) | Argentina, Brazil, Paraguay | NT |
|  | *Pteroglossus castanotis*  (Chestnut-eared Aracari) | 2 (7) | South America | LC |
|  | *Ramphastos dicolorus*  (Red-breasted Toucan) | 4 (14) | Argentina, Brazil, Paraguay | LC |
|  | *Ramphastos tucanus*  (Red-billed Toucan) | 4 (10) | South America | VU |
|  | *Selenidera maculirostris*  (Spot-billed Toucanet) | 5 (17) | Argentina, Brazil, Paraguay, Bolivia | LC |
| PSITTACIFORMES |  |  |  |  |
| Cacatuidae | *Cacatua moluccensis*  (Salmon-crested Cockatoo) ∆ | 1 (15) | Indonesia | VU |
|  | *Nymphicys hollandicus*  (Cockatiel) ∆ | 1 (1) | Australia | LC |
| Psittacidae | *Amazona amazonica*  (Orange-winged Parrot) | 2 (2) | South America | LC |
|  | *Amazona brasiliensis*  (Red-tailed Parrot) | 9 (9) | Brazil | VU |
|  | *Amazona farinosa*  (Mealy Parrot) | 2 (5) | South America | NT |
|  | *Amazona festiva*  (Festive Parrot) | 6 (6) | Brazil, Colombia, Ecuador, Peru | NT |
|  | *Amazona ochrocephala*  (Yellow-crowned Parrot) | 4 (7) | South America | LC |
|  | *Amazona rhodocorytha*  (Red-browed Parrot) | 3 (3) | Brazil | EM |
|  | *Amazona* sp.  (Parrot) | 2 (2) | - | - |
|  | *Amazona vinacea*  (Vinaceous-breasted Parrot) | 3 (6) | Argentina, Brazil, Paraguay | EM |
|  | *Anodorhynchus leari*  (Indigo Macaw) | 15 (44) | Brazil | EM |
|  | *Ara ararauna*  (Blue-and-yellow Macaw) | 3 (3) | South America | LC |
|  | *Ara chloropterus*  (Red-and-green Macaw) | 3 (10) | South America | LC |
|  | *Ara macao*  (Scarlet Macaw) | 3 (12) | South and Central America | LC |
|  | *Ara rubrogenys*  (Red-fronted Macaw) ∆ | 5 (8) | Bolivia | EN |
|  | *Ara severus*  (Chestnut fronted Macaw) | 2 (2) | South America | LC |
|  | *Aratinga auricapillus*  (Golden-capped Parakeet) | 1 (1) | Brazil | NT |
|  | *Aratinga jandaya*  (Jandaya Parakeet) | 2 (3) | Brazil | LC |
|  | *Aratinga nenday*  (Nanday Parakeet) | 9 (17) | Argentina, Bolivia, Brazil, Paraguay | LC |
|  | *Aratinga solstitialis*  (Sun Parakeet) | 2 (2) | Brazil, Guyana | EN |
|  | *Deroptyus accipitrinus*  (Red-fan Parrot) | 3 (9) | South America | LC |
|  | *Eos bornea*  (Red Lory) ∆ | 2 (5) | Indonesia | LC |
|  | *Eupsittula aurea*  (Peach-fronted Parakeet) | 15 (16) | Argentina, Bolivia, Brazil, Paraguay, Peru, Suriname | LC |
|  | *Graydidascalus brachyurus*  (Short-tailed Parrot) | 1 (2) | Brazil, Colombia, Ecuador, French Guyana, Peru | LC |
|  | *Pionites leucogaster*  (White-bellied Parrot) | 6 (6) | Brazil | EN |
|  | *Pionites melanocephalus*  (Black-headed Parrot) | 2 (2) | South America | LC |
|  | *Pionopsitta pileata*  (Pileated Parrot) | 1 (1) | Argentina, Brazil, Paraguay | LC |
|  | *Pionus fuscus*  (Dusky Parrot) | 3 (3) | North of South America | LC |
|  | *Pionus menstruus*  (Blue-headed Parrot) | 4 (8) | North of South America | LC |
|  | *Poicephalus senegalus*  (Senegal Parrot) ∆ | 2 (3) | Africa | LC |
|  | *Primolius auricollis*  (Yellow-collared Macaw) | 2 (2) | Argentina, Bolivia, Brazil, Paraguay | LC |
|  | *Primolius maracana*  (Blue-winged Macaw) | 2 (6) | Argentina, Brazil, Paraguay | NT |
|  | *Psittacara leucophthalmus*  (White-eyed Parakeet) | 3 (5) | South America | LC |
|  | *Pyrrhura frontalis*  (Maroon-bellied Parakeet) | 2 (4) | Argentina, Brazil, Paraguay, Uruguay | LC |
|  | *Pyrrhura lepida lepida*  (Pearly Parakeet) | 3 (3) | Brazil | VU |
|  | *Thectocercus acuticaudatus*  (Blue-crowned Parakeet) | 2 (3) | South of South America | LC |
| RHEIFORMES |  |  |  |  |
| Rheidae | *Rhea americana*  (Greater Rhea) | 4 (6) | Argentina, Bolivia, Brazil, Paraguay, Uruguay | NT |
| STRIGIFORMES |  |  |  |  |
| Strigidae | *Asio stygius*  (Stygian Owl) | 3 (5) | South America | LC |
|  | *Bubo virginianus*  (Great Horned Owl) | 1 (2) | South and Central America | LC |
|  | *Megascops choliba*  (Tropical Screech-Owl) | 2 (2) | South America | LC |
|  | *Pseudoscops clamator*  (Striped Owl) | 2 (18) | South and Central America | LC |
|  | *Pulsatrix perspicillata*  (Spectacled Owl) | 2 (6) | South and Central America | LC |
| Tytonidae | *Tyto alba*  (Barn Owl) | 3 (5) | South and Central America, United States, Africa, Europe, Oceania, South of Asia | LC |

IUCN Threatened status: least concern (LC), near threatened (NT), vulnerable (VU), endangered (EN), critically endangered (CR). ∆ Not native to Brazil.
